# Higher education students’ perceptions of ChatGPT: A global study of early reactions

**DOI:** 10.1371/journal.pone.0315011

**Published:** 2025-02-05

**Authors:** Dejan Ravšelj, Damijana Keržič, Nina Tomaževič, Lan Umek, Nejc Brezovar, Noorminshah A. Iahad, Ali Abdulla Abdulla, Anait Akopyan, Magdalena Waleska Aldana Segura, Jehan AlHumaid, Mohamed Farouk Allam, Maria Alló, Raphael Papa Kweku Andoh, Octavian Andronic, Yarhands Dissou Arthur, Fatih Aydın, Amira Badran, Roxana Balbontín-Alvarado, Helmi Ben Saad, Andrea Bencsik, Isaac Benning, Adrian Besimi, Denilson da Silva Bezerra, Chiara Buizza, Roberto Burro, Anthony Bwalya, Cristina Cachero, Patricia Castillo-Briceno, Harold Castro, Ching Sing Chai, Constadina Charalambous, Thomas K. F. Chiu, Otilia Clipa, Ruggero Colombari, Luis José H. Corral Escobedo, Elísio Costa, Radu George Crețulescu, Marta Crispino, Nicola Cucari, Fergus Dalton, Meva Demir Kaya, Ivo Dumić-Čule, Diena Dwidienawati, Ryan Ebardo, Daniel Lawer Egbenya, MoezAlIslam Ezzat Faris, Miroslav Fečko, Paulo Ferrinho, Adrian Florea, Chun Yuen Fong, Zoë Francis, Alberto Ghilardi, Belinka González-Fernández, Daniela Hau, Md. Shamim Hossain, Theo Hug, Fany Inasius, Maryam Jaffar Ismail, Hatidža Jahić, Morrison Omokiniovo Jessa, Marika Kapanadze, Sujita Kumar Kar, Elham Talib Kateeb, Feridun Kaya, Hanaa Ouda Khadri, Masao Kikuchi, Vitaliy Mykolayovych Kobets, Katerina Metodieva Kostova, Evita Krasmane, Jesus Lau, Wai Him Crystal Law, Florin Lazăr, Lejla Lazović-Pita, Vivian Wing Yan Lee, Jingtai Li, Diego Vinicio López-Aguilar, Adrian Luca, Ruth Garcia Luciano, Juan D. Machin-Mastromatteo, Marwa Madi, Alexandre Lourenço Manguele, Rubén Francisco Manrique, Thumah Mapulanga, Frederic Marimon, Galia Ilieva Marinova, Marta Mas-Machuca, Oliva Mejía-Rodríguez, Maria Meletiou-Mavrotheris, Silvia Mariela Méndez-Prado, José Manuel Meza-Cano, Evija Mirķe, Alpana Mishra, Ondrej Mital, Cristina Mollica, Daniel Ionel Morariu, Natalia Mospan, Angel Mukuka, Silvana Guadalupe Navarro Jiménez, Irena Nikaj, Maria Mihaylova Nisheva, Efi Nisiforou, Joseph Njiku, Singhanat Nomnian, Lulzime Nuredini-Mehmedi, Ernest Nyamekye, Alka Obadić, Abdelmohsen Hamed Okela, Dorit Olenik-Shemesh, Izabela Ostoj, Kevin Javier Peralta-Rizzo, Almir Peštek, Amila Pilav-Velić, Dilma Rosanda Miranda Pires, Eyal Rabin, Daniela Raccanello, Agustine Ramie, Md. Mamun ur Rashid, Robert A. P. Reuter, Valentina Reyes, Ana Sofia Rodrigues, Paul Rodway, Silvia Ručinská, Shorena Sadzaglishvili, Ashraf Atta M. S. Salem, Gordana Savić, Astrid Schepman, Samia Mokhtar Shahpo, Abdelmajid Snouber, Emma Soler, Bengi Sonyel, Eliza Stefanova, Anna Stone, Artur Strzelecki, Tetsuji Tanaka, Carolina Tapia Cortes, Andrea Teira-Fachado, Henri Tilga, Jelena Titko, Maryna Tolmach, Dedi Turmudi, Laura Varela-Candamio, Ioanna Vekiri, Giada Vicentini, Erisher Woyo, Özlem Yorulmaz, Said A. S. Yunus, Ana-Maria Zamfir, Munyaradzi Zhou, Aleksander Aristovnik

**Affiliations:** 1 Faculty of Public Administration, University of Ljubljana, Ljubljana, Slovenia; 2 Department of Information Systems, Faculty of Management, Universiti Teknologi Malaysia, Skudai, Johor Bahru, Malaysia; 3 Department of Computer Science and IT, State University of Zanzibar (SUZA), Zanzibar, Tanzania; 4 Department of English for the Humanities, Southern Federal University, Rostov-on-Don, Russia; 5 Education Department, Galileo University, Guatemala, Guatemala; 6 Physics Department, San Carlos de Guatemala University, Guatemala, Guatemala; 7 Department of Preventive Dental Sciences, College of Dentistry, Imam Abdulrahman Bin Faisal University, Dammam, Saudi Arabia; 8 Department of Family Medicine, Ain Shams University, Cairo, Egypt; 9 Department of Economics, Faculty of Economics and Business, University of A Coruna, A Coruna, Spain; 10 Directorate of Research, Innovation and Consultancy, University of Cape Coast, Cape Coast, Ghana; 11 Innovation and eHealth Center, Carol Davila University of Medicine and Pharmacy, Bucharest, Romania; 12 Department of Mathematics Education, Faculty of Applied Sciences and Mathematics Education, Akenten Appiah Menka University of Skills Training and Entrepreneurial Development (AAMUSTED), Kumasi, Ghana; 13 Faculty of Education, Sivas Cumhuriyet University, Sivas, Türkiye; 14 Faculty of Dentistry, Ain Shams University, Cairo, Egypt; 15 Faculty of Education and Humanities, University of Bío Bío, Chillán, Chile; 16 Research Laboratory LR12SP09 “Heart Failure”, Faculty of Medicine of Sousse, University of Sousse, Sousse, Tunisia; 17 Department of Management, University of Pannonia, Veszprem, Hungary; 18 Department of Management, J. Selye University, Komarno, Slovakia; 19 Department of Mathematics & ICT Education, University of Cape Coast, Cape Coast, Ghana; 20 Faculty of Contemporary Sciences and Technologies, South East European University, Tetovo, Republic of North Macedonia; 21 Department of Oceanography and Limnology. Federal University of Maranhão, São Luis, Brazil; 22 Department of Clinical and Experimental Sciences, University of Brescia, Brescia, Italy; 23 Department of Human Sciences, University of Verona, Verona, Italy; 24 Department of Biological Sciences, Kwame Nkrumah University, Kitwe, Zambia; 25 Languages and Computer Systems, University of Alicante, Alicante, Spain; 26 EBIOAC Lab, Faculty of Life Sciences and Technologies, Universidad Laica Eloy Alfaro de Manabi ULEAM, Manta, Ecuador; 27 Department of Systems and Computing Engineering, Universidad de los Andes, Bogota, Colombia; 28 Centre for Learning Sciences and Technologies, Chinese University of Hong Kong, Hong Kong, Hong Kong SAR, China; 29 Department of Education, European University Cyprus, Nicosia, Cyprus; 30 Department of Curriculum and Instruction, Chinese University of Hong Kong, Hong Kong, Hong Kong SAR, China; 31 Science of Education, Stefan cel Mare University of Suceava, Suceava, Romania; 32 Department of Economics and Social Sciences, International University of Catalonia, Barcelona, Spain; 33 IAM, Physics Department, CUCEI, University of Guadalajara, Guadalajara, Mexico; 34 Competence Center on Active and Healthy Ageing and CINTESIS@Rise, Faculty of Pharmacy, University of Porto, Porto, Portugal; 35 Computer Science and Electrical Engineering Department, Faculty of Engineering, Lucian Blaga University of Sibiu, Sibiu, Romania; 36 Independent Researcher, Rome, Italy; 37 Department of Management, Faculty of Economics, Sapienza University of Rome, Rome, Italy; 38 Department of Psychology, University of the Fraser Valley, Abbotsford, Canada; 39 Faculty of Letters, University of Ataturk, Erzurum, Turkiye; 40 Department of Nursing, University North, Varaždin, Croatia; 41 BINUS Business School, Bina Nusantara University, Jakarta, Indonesia; 42 Department of Information Technology, De La Salle University, Manila, Philippines; 43 College of Health and Allied Sciences, University of Cape Coast, Cape Coast, Ghana; 44 Faculty of Allied Medical Sciences, Applied Sciences Private University, Jordan, Jordan; 45 Faculty of Public Administration, Pavol Jozef Šafárik University in Košice, Košice, Slovakia; 46 Global Health and Tropical Medicine (GHTM), Associate Laboratory in Translation and Innovation Towards Global Health (LA-REAL), Instituto de Higiene e Medicina Tropical (IHMT), Nova University of Lisbon, Lisbon, Portugal; 47 International College of Liberal Arts, Yamanashi Gakuin University, Kofu, Japan; 48 Department of Sciences and Engineering, Ibero-American University Puebla, Puebla, Mexico; 49 Department of Education and Social Work, University of Luxembourg, Belval Esch-sur-Alzette, Luxembourg; 50 Department of Marketing, Hajee Mohammad Danesh Science and Technology University, Dinajpur, Bangladesh; 51 Department of Media, Society and Communication, University of Innsbruck, Innsbruck, Austria; 52 School of Accounting, Bina Nusantara University, Jakarta, Indonesia; 53 School of Education, State University of Zanzibar (SUZA), Zanzibar, Tanzania; 54 School of Economics and Business, University of Sarajevo, Sarajevo, Bosnia and Herzegovina; 55 Guidance and Counselling, Delta State University Abraka, Abraka, Nigeria; 56 School of Business Technology and Education, Ilia State University, Tbilisi, Georgia; 57 Department of Psychiatry, King George’s Medical University, Lucknow, India; 58 Oral Health Research and Promotion Unit, Al-Quds University. Jerusalem, Palestine; 59 Faculty of Education, Ain Shams University, Cairo, Egypt; 60 Department of Public Management. Meiji University, Tokyo, Japan; 61 Computer Science and Software Engineering Department, Kherson State University, Kherson, Ukraine; 62 Department of Technologies and Management of Communication Systems, Faculty of Telecommunications, Technical University of Sofia, Sofia, Bulgaria; 63 Department of Education, Alberta College, Riga, Latvia; 64 Faculty of Pedagogy, Universidad Veracruzana, Veracruz, Mexico; 65 Sociology and Social Work, University of Bucharest, Bucharest, Romania; 66 Centre for Learning Enhancement and Research (CLEAR), Chinese University of Hong Kong, Shatin, Hong Kong SAR, China; 67 School of Foreign Languages, Jiaying University, Meizhou, China; 68 Languages Department, Indoamerica Technological University, Ambato, Ecuador; 69 Department of Applied Psychology and Psychotherapy, Faculty of Psychology and Educational Sciences, University of Bucharest, Bucharest, Romania; 70 College of Information and Communications Technology, Nueva Ecija University of Science and Technology, Cabanatuan City, Nueva Ecija, Philippines; 71 Faculty of Philosophy and Letters, Autonomous University of Chihuahua, Chihuahua, Mexico; 72 Health Sciences Department, Higher Institute of Health Sciences, Maputo, Mozambique; 73 African Centre of Excellence for Innovative Teaching and Learning Mathematics and Science, University of Rwanda, Kayonza, Rwanda; 74 Medicine School, Vasco de Quiroga University, Morelia, Mexico; 75 Faculty of Social Sciences and Humanities, ESPOL Polytechnic University, Guayaquil, Ecuador; 76 Faculty of Higher Education Iztacala, National Autonomous University of Mexico, State of Mexico, Mexico; 77 Institute of Digital Humanities, Faculty of Computer Science, Information Technology and Energy, Riga Technical University, Riga. Latvia; 78 Department of Community Medicine, Kalinga Institute of Medical Science. Bhubaneswar, India; 79 Department of Statistical Sciences, Sapienza University of Rome, Rome, Italy; 80 Department of Linguistics and Translation, Borys Grinchenko Kyiv University, Kyiv, Ukraine; 81 Department of Mathematics, Science and Technology Education, Mukuba University, Kitwe, Zambia; 82 Faculty of Education & Philology, University Fan S. Noli Korça, Korça, Albania; 83 Faculty of Mathematics and Informatics, Sofia University St. Kliment Ohridski, Sofia, Bulgaria; 84 Department of Education, University of Nicosia, Nicosia, Cyprus; 85 Educational Psychology and Curriculum Studies, Dar es Salaam University College of Education, University of Dar es Salaam, Dar es Salaam, Tanzania; 86 Research Institute for Languages and Cultures of Asia, Mahidol University, Salaya, Thailand; 87 Data Analysis Office, South East European University, Tetovo, Republic of North Macedonia; 88 Department of Arts Education, University of Cape Coast, Cape Coast, Ghana; 89 Faculty of Economics and Business, University of Zagreb, Zagreb, Croatia; 90 Faculty of Specific Education, Minia University, Minia, Egypt; 91 Education & Psychology, The Research Center for Innovation in Learning Technologies, Open University of Israel, Raanana, Israel; 92 Department of Economics, Faculty of Economics, University of Economics in Katowice, Katowice, Poland; 93 Faculty of Science and Technology, University of Cape Verde, Praia, Cape Verde; 94 Nursing Department, Health Polytechnic of Banjarmasin, Banjarbaru, Indonesia; 95 Department of Agricultural Extension and Rural Development, Patuakhali Science and Technology University, Patuakhali, Bangladesh; 96 Facultad de Economía y Negocios, Universidad de Chile, Santiago, Chile; 97 Polytechnic Institute of Viana do Castelo, Viana do Castelo, Portugal; 98 Division of Psychology, Faculty of Health, Medicine and Society, University of Chester, Chester, United Kingdom; 99 Research Center for Advancing Science in the Social Services and Interventions, Social Work Program, Faculty of Arts and Science, Ilia State University, Tbilisi, Georgia; 100 College of Languages & Translation, Sadat Academy for Management Sciences, Alexandria, Egypt; 101 Faculty of Organizational Sciences, University of Belgrade, Belgrade, Serbia; 102 Department of Early Childhood, College of Sciences and Humanities Studies, Imam Abdulrahman Bin Faisal University, Dammam, Saudi Arabia; 103 Faculty of Medicine, University of Oran1, Oran, Algeria; 104 Department of Educational Sciences, Eastern Mediterranean University, Famagusta, Cyprus; 105 School of Psychology, University of East London, London, United Kingdom; 106 Department of Informatics, University of Economics in Katowice, Katowice, Poland; 107 Department of Economics, Meiji Gakuin University, Tokyo, Japan; 108 Department of Education and Humanities, University of Monterrey, Monterrey, Mexico; 109 Public Law, Faculty of Law, University of A Coruna, A Coruna, Spain; 110 Institute of Sport Sciences and Physiotherapy, University of Tartu, Tartu. Estonia; 111 EKA University of Applied Sciences, Riga, Latvia; 112 Faculty of Distance Learning, Kyiv National University of Culture and Arts, Kyiv, Ukraine; 113 English Education Study Program, Faculty of Teachers’ Training and Education, Muhammadiyah University of Metro, Metro, Indonesia; 114 Faculty of Business & Law, Manchester Metropolitan University, Manchester, United Kingdom; 115 Department of Econometrics & Statistics, Faculty of Economics, Istanbul University, Istanbul, Türkiye; 116 Faculty of Business and Administration, University of Bucharest, Bucharest, Romania; 117 National Scientific Research Institute for Labour and Social Protection, Bucharest, Romania; 118 Information and Marketing Sciences, Midlands State University, Gweru, Zimbabwe; East China Normal University, CHINA

## Abstract

The paper presents the most comprehensive and large-scale global study to date on how higher education students perceived the use of ChatGPT in early 2024. With a sample of 23,218 students from 109 countries and territories, the study reveals that students primarily used ChatGPT for brainstorming, summarizing texts, and finding research articles, with a few using it for professional and creative writing. They found it useful for simplifying complex information and summarizing content, but less reliable for providing information and supporting classroom learning, though some considered its information clearer than that from peers and teachers. Moreover, students agreed on the need for AI regulations at all levels due to concerns about ChatGPT promoting cheating, plagiarism, and social isolation. However, they believed ChatGPT could potentially enhance their access to knowledge and improve their learning experience, study efficiency, and chances of achieving good grades. While ChatGPT was perceived as effective in potentially improving AI literacy, digital communication, and content creation skills, it was less useful for interpersonal communication, decision-making, numeracy, native language proficiency, and the development of critical thinking skills. Students also felt that ChatGPT would boost demand for AI-related skills and facilitate remote work without significantly impacting unemployment. Emotionally, students mostly felt positive using ChatGPT, with curiosity and calmness being the most common emotions. Further examinations reveal variations in students’ perceptions across different socio-demographic and geographic factors, with key factors influencing students’ use of ChatGPT also being identified. Higher education institutions’ managers and teachers may benefit from these findings while formulating the curricula and instructions/regulations for ChatGPT use, as well as when designing the teaching methods and assessment tools. Moreover, policymakers may also consider the findings when formulating strategies for secondary and higher education system development, especially in light of changing labor market needs and related digital skills development.

## Introduction

Artificial Intelligence (AI), originating in the 1950s, began as an exploration of machines mimicking human behavior and cognition [1, 2]. This pursuit led to diverse fields like machine learning, natural language processing (NLP), computer vision, and robotics, each advancing AI’s capacity to emulate human reasoning, learning, and discernment [3]. To date, ChatGPT stands out as the most popular interactive generative AI model based on Natural Language Processing (NLP), a field of AI that enables computers to understand, interpret, and generate human language. It leverages Large Language Models (LLMs), which are advanced algorithms trained on vast amounts of text data to generate human-like responses and perform a wide range of language tasks with high accuracy and versatility [4, 5].

Developed by OpenAI, a globally recognized AI research organization, a conversational chatbot ChatGPT was first released on November 30, 2022, with the primary objectives of enhancing human-AI interaction and expanding AI applications in practical domains [6]. As a fine-tuned GPT model for conversational tasks, ChatGPT is freely accessible on multiple platforms, enabling users to interact seamlessly for conversations, answers, and content creation in various styles and languages [7–9]. Due to the wide possibilities of ChatGPT’s use, teachers and students in higher education also started using ChatGPT immediately after its release. At the same time, many researchers became curious about what ChatGPT can and cannot do, how students interact with ChatGPT for better learning results, and how teachers collaborate with ChatGPT within their teaching methods [10–13]. In our research, we focused only on students’ perspectives, which will be elaborated further in the paper.

ChatGPT offers significant applications in higher education by providing continuous, on-demand support, personalized tutoring, enhanced revision tools, and accessibility aid, especially benefiting students who require flexible learning options, customized explanations, or language assistance. Additionally, its capabilities for generating practice questions, summarizing content, and assisting in academic writing make it a valuable tool for student learning and self-directed study. However, these advantages are accompanied by challenges, including concerns about academic integrity due to the potential for misuse in assignments and exams, the risk of overreliance that may hinder critical thinking development, and occasional inaccuracies in responses that can mislead students lacking strong foundational knowledge. Privacy and data security concerns add to the complexity, as students are unsure about data handling practices, and the AI’s lack of emotional and contextual understanding limits its effectiveness in addressing the nuanced needs of learners. Furthermore, ChatGPT’s quick, compartmentalized answers risk promoting fragmented learning rather than fostering comprehensive conceptual understanding, underscoring the need for a balanced integration of AI that encourages ethical use while preserving the educational rigor and integrity essential in higher education [14].

Therefore, ChatGPT has the potential to foster or hinder students’ learning. It can reduce cognitive load by handling complex tasks, freeing up working memory and supporting critical thinking, but it may also lead to dependency and cognitive overload by replacing deep thinking [15]. For instance, some students could use ChatGPT’s diverse writing and learning assistance capabilities to support self-regulated learning (SRL) by aiding in goal-setting and preparation (forethought), promoting active engagement through note-taking, question preparation and practice (performance), and enhancing comprehension through self-assessment and peer discussions (self-reflection) [16]. Others, however, might use it to complete academic assignments without developing a critical understanding of the task or engaging in meaningful learning [17]. The effectiveness of learning how to use ChatGPT thus depends on the students’ AI competency [18, 19]. Students with strong AI competency have the confidence, knowledge, and skills to apply ChatGPT effectively and responsibly, leveraging it for new perspectives and feedback to enhance their learning. This competency involves not only understanding the capabilities of ChatGPT but also recognizing its limitations, making informed and ethical decisions on its use, and interpreting AI-generated content critically to ensure meaningful learning outcomes. Moreover, students’ learning is significantly influenced by instructional design and assessment approaches. This dual role of teachers involves developing assessment designs that integrate AI tools like ChatGPT for educational purposes and guiding students in using these tools responsibly, effectively, and ethically. The ability of teachers to create AI-supported assessments and instruct students on best practices in AI usage plays a critical role in shaping students’ learning experiences [20, 21]. As students will likely interact with AI tools like ChatGPT in their future careers, building their confidence and competence in using AI is essential for their professional development.

### Research objectives

The main goal of the paper is to present a comprehensive global study on higher education students’ perceptions of different aspects of ChatGPT use related to their study and career development challenges. The study focuses on various aspects of ChatGPT, including its usage and capabilities, regulation and ethical concerns, satisfaction with and attitudes towards ChatGPT, study issues and outcomes associated with its use, skills development, labor market and skills mismatch, and emotions related to the use of ChatGPT. The main purpose of our research was to explore the perspectives of students worldwide on ChatGPT and to propose recommendations for higher education teachers, managers, and policymakers regarding curriculum design, diverse teaching and assessment methods, and regulations and strategies to support effective AI integration in education.

While the release of ChatGPT has garnered significant attention from educators globally, there have been only a few attempts in the literature to explore students’ perceptions of ChatGPT in higher education, capturing the perspective of students from different countries and regions. Analyzing Twitter data from over 16 million tweets and more than 5.5 million users, Fütterer et al. [22] explored global perceptions and reactions to ChatGPT in the context of education, revealing that education was the most frequently tweeted topic related to ChatGPT. However, there were also attempts to examine this topic through student surveys. Bouteraa et al. [23] recruited 921 students from Asian countries to explore ChatGPT usage, capabilities, satisfaction, attitudes, study issues, skills development, and personal anxiety, although they neglected other important issues such as regulation, ethical concerns, labor market implications, and broader emotional responses. Ibrahim et al. [24] recruited 1,601 students from large countries, including Brazil, India, Japan, the United Kingdom, and the United States, focusing on ChatGPT usage, capabilities, satisfaction, and attitude, but omitted regulation, skills development, labor market implications, and emotional responses. Abdaljaleel et al. [25] recruited 2,240 students from Arabic countries, offering a more comprehensive view on ChatGPT usage and capabilities but only briefly addressing labor market implications and skills mismatch. Our study fills the research gap by providing a global perspective on students’ perceptions of ChatGPT, covering a wide range of aspects. Accordingly, the following research question was formulated:

**RQ1:** How do students perceive different aspects of ChatGPT related to its usage, capabilities, regulation and ethical concerns, satisfaction and attitude, study issues and outcomes, skills development, labor market and skills mismatch, and emotional responses?

Despite the growing body of research on students’ perceptions of ChatGPT, studies specifically examining the impact of socio-demographic characteristics remain limited and inconclusive. Some researchers suggest that students’ perceptions of ChatGPT are influenced by factors such as country of residence, age, type of university, and recent academic performance [25]. However, other research indicates that perceptions of ChatGPT usage among higher education students do not significantly differ based on gender, academic programs, or educational streams [26]. Some research has even found mixed results regarding the moderating effect of gender and study level on the acceptance and use of generative AI by higher education students [27]. None of these studies, however, have yet addressed the importance of academic discipline (i.e., field of study) and income regions in the context of students’ experiences with ChatGPT. Accordingly, the following research question was formulated:

**RQ2:** How do students’ perceptions of various ChatGPT aspects differ across fields of study, income regions, and other selected socio-demographic and geographic characteristics, including gender, level of study, mode of study, area of living, and economic status?

The acceptance and use of ChatGPT have been relatively well documented, revealing that performance expectancy, effort expectancy, social influence, and facilitating conditions significantly influence behavioral intention and actual use across diverse educational contexts [28]. However, it remains unclear how various factors related to ChatGPT influence students’ usage patterns, especially due to the lack of empirical analysis on a large global scale. Accordingly, the following research question was formulated:

**RQ3:** How do selected factors related to ChatGPT aspects influence students’ usage patterns of ChatGPT for various tasks, including brainstorming, summarizing, and academic writing?

The adoption of ChatGPT in higher education presents both opportunities and challenges for students. While it enhances learning, engagement, and skills development, it also raises concerns about academic integrity, emotional impacts, and labor market implications. To maximize benefits and mitigate drawbacks, it is crucial to understand student perceptions from all these perspectives. Feedback from students, particularly early adopters who are typically the most enthusiastic and influential users, provides valuable insights for the effective and responsible integration of ChatGPT into higher education, ultimately enhancing the overall learning experience.

## Literature review

The existing literature offers valuable insights into eight aspects of ChatGPT, including usage, capabilities, regulatory and ethical concerns, user satisfaction and attitudes, academic issues and outcomes, skills development, labor market dynamics and skills mismatches, and emotional responses. These topics are briefly outlined below, providing a foundation for a more in-depth exploration of each specific aspect.

Research on ChatGPT highlights its potential to enhance learning [2]. Students’ adoption of ChatGPT is influenced by factors beyond ease of use, including perceived usefulness, social presence, the tool’s legitimacy, enjoyment, and motivation [29]. Engaging with functionalities that improve learning are particularly valued. Several studies highlight the potential of ChatGPT *usage* in higher education, especially for supporting assessment preparation, translation, linguistic training, argumentative writing, research and analysis, programming, and scientific writing. In assessment preparation, ChatGPT offers interactive problem-solving and explanations, helping students better understand complex concepts. It supports translation and linguistic training by providing real-time feedback on grammar and vocabulary, which is valuable in multilingual settings. For argumentative writing, ChatGPT assists in structuring and refining arguments, while in research, it helps with organizing ideas and synthesizing information. In programming, it offers debugging and coding tips, fostering skill development, and in scientific writing, it guides students in adhering to formal conventions and managing citations. These applications showcase ChatGPT’s adaptability in enhancing personalized learning across diverse academic areas [30–36].

Regarding its *capabilities*, ChatGPT is a versatile tool that enhances the learning experience in higher education by understanding and responding in human language, providing clear explanations, simplifying complex topics, and offering structured guidance. Acting as both a virtual peer and an assessor, ChatGPT supports critical thinking, resource identification, and content refinement, complementing both traditional classroom and online learning environments [31, 37, 38]. Its conversational abilities foster engagement and enable it to bridge digital and in-person learning, making it a valuable partner in blended (hybrid) learning [29]. However, recognizing its limitations, such as potential misinformation, untested response accuracy, data quality issues, and ethical considerations, is essential for maximizing its positive impact on student learning outcomes [2, 32, 39–42]. By addressing these challenges, educators and students can leverage ChatGPT to support a more personalized and efficient educational journey [42, 43].

The presence of ChatGPT has also sparked *regulation and ethical concerns* surrounding academic integrity in higher education. Most higher education institutions lack rules for its use. Despite the controversies surrounding ChatGPT’s impact, stakeholders view it as an opportunity to enhance student learning and access. This perspective is evident from workshops focused on using ChatGPT legally and avoiding plagiarism in scientific writing. Opinions on generative AI tools like ChatGPT are divided: some emphasize the increased ease and comfort in learning, while others express concerns about potential cheating and academic integrity issues if regulations are not established [9]. Further research and discussion on the implications of AI tools, ethical use in scientific writing, and innovative teaching practices are needed. Some institutions have banned ChatGPT from writing articles and research due to concerns about academic integrity, content bias, and ethical issues related to patient privacy, as well as the need for thorough screening before widespread adoption, particularly in clinical research and medical practice [44–46]. Studies addressing ethical concerns and regulation highlight the necessity of minimizing risks and unintended consequences while integrating AI into education through ongoing dialogue and research [47].

In general, students report high *satisfaction and* positive *attitude* with ChatGPT for its instant, detailed responses and assistance in understanding complex topics [48, 49]. Higher knowledge and positive attitudes correlate with increased ChatGPT usage, especially among final-level students [50]. Satisfaction arises from the perceived efficiency and personalized learning experiences that these technologies offer. ChatGPT also enhances research capabilities, contributing to academic satisfaction through quicker access to scholarly materials. However, concerns about the reliability and accuracy of ChatGPT’s information persist, necessitating cross-verification and cautious use [51]. Computer science students suggest clearer guidelines for effective utilization [52]. Additionally, ChatGPT’s inability to respond to emotional cues limits its effectiveness compared to human tutors [48–54]. These mixed feelings underscore the need for guidelines and training on the ethical use of AI tools. While higher education students generally have a positive attitude towards ChatGPT, educators must address ethical concerns and the risk of dependency. Integrating AI tools responsibly into the curriculum can maximize benefits while mitigating drawbacks, and effective use should be complemented by the development of critical thinking and information literacy skills.

Research suggests that ChatGPT can effectively support students in addressing *study issues and* improving *learning outcomes*, with evidence showing a positive correlation between its use and enhanced academic performance. This positive impact is driven particularly by the personalized feedback and interactive experiences AI chatbots provide, which have proven to boost motivation, engagement, and self-efficacy while reducing learning anxiety [55]. Students report significant improvements in academic performance due to quick responses and relevant resources, especially outside regular classroom hours [56], as well as immediate assistance for academic tasks [57]. Students in scientific disciplines particularly noted its role in enhancing their understanding of complex subjects [48]. Responses to ChatGPT vary across cultural and linguistic backgrounds, necessitating nuanced approaches to its use [58]. To ensure the effective use of ChatGPT, students should embrace emerging technologies while being trained to apply AI outputs responsibly, avoiding overreliance or academic misconduct. Clear guidance on AI misuse, aligned with teaching information literacy, is crucial [53, 59]. Ngo [56] suggests that reducing integrity risks, such as assessing information quality and citing sources accurately, can improve study outcomes. Strategies like verifying responses, providing clear usage guidelines, and promoting academic integrity are essential for the ethical use of AI in academia. Javaid et al. [60] highlight ChatGPT’s potential in tutoring and personalized learning. However, balancing the convenience of AI with encouraging independent application of knowledge and skills is essential for effective integration into educational contexts.

Research shows ChatGPT enhances *skills development* by offering an interactive environment and extensive knowledge [61, 62]. Lee [33] found that its use in medical education creates interactive virtual learning environments, improving learning and communication skills [63–66]. ChatGPT also enhances students’ writing skills by providing real-time grammar correction, vocabulary enrichment, and compositional feedback [67–69]. Effective incorporation into education requires balancing AI assistance with fostering intrinsic writing skills [9, 70]. Engaging higher education students in problem-solving tasks improves knowledge transfer, critical thinking, and idea generation [71–73]. Studies show that smart personal assistants support problem-solving skills [74, 75]. Essien et al. [76] suggest that AI-driven learning promotes critical thinking and encourages new teaching methods. Urban et al. [77] found that ChatGPT improves complex problem-solving performance and self-efficacy but has an unclear impact on task interest and metacognitive monitoring. Concerns exist about AI disrupting problem-solving processes by generating diverse solutions [78–80], but evidence regarding ChatGPT’s impact on actual performance is limited [81].

Studying *labor market and skills mismatch* issues related to ChatGPT in higher education is crucial [82]. Chen et al. [83] suggest that jobs involving writing and programming are more susceptible to replacement by language models, with higher-paying positions at greater risk. They conclude that learning ChatGPT skills offers a competitive advantage in the labor market. ChatGPT can simulate human thinking, understand and generate language, and assist in task completion more efficiently. In China, 28% of occupations require ChatGPT skills, especially in high-paying industries like technology and sales, where algorithm engineers benefit from improved efficiency [83, 84]. However, Shoufan [85] claims that senior computer engineering students view ChatGPT as a job threat but notes that the perceived negative impact of ChatGPT on job opportunities is moderate, aligning with its role as a complementary tool. Despite this, ChatGPT is seen as a complementary resource rather than a replacement for human intelligence, prompting universities to integrate it into curricula [86–88]. Huseynov [89] finds that US students exhibit pessimistic shifts after negative ChatGPT discussions, with female students particularly concerned. Addressing skills mismatches is critical, and AI like ChatGPT can help by identifying skill gaps and recommending targeted training [84, 90].

Adopting new technologies like ChatGPT elicits both positive and negative *emotions* in students. Under significant academic pressure, students are more inclined to use ChatGPT for its immediate unburdening effect [91]. A study by Hadi Mogavi et al. [92] found mixed emotions among early adopters in education: excitement about its benefits and convenience, alongside apprehension about dependency. Mamo et al. [93] analyzed social media posts from higher education faculty and found mostly neutral (51%), positive (40%), and some negative (9%) sentiments. Trust and joy were common positive emotions, while anger and fear were the primary negative ones. Fütterer et al. [22] revealed that emotional reactions to ChatGPT are heterogeneous and change over time. AI significantly reduces work burdens, making academic tasks easier, but it may potentially affect academic integrity [94]. ChatGPT use may also cause memory-related issues and procrastination, negatively impacting academic performance and increasing stress and anxiety [91, 95]. This highlights the need for cautious and responsible use of AI tools like ChatGPT.

While the current literature provides valuable insights into various aspects of ChatGPT use in higher education, several critical research gaps remain. First, although existing studies explore ChatGPT’s impact on learning outcomes, skill development, and ethical concerns, they often lack a global perspective that captures diverse cultural, economic, and educational contexts. This limitation reduces the generalizability of findings and overlooks how regional variations shape students’ experiences with ChatGPT. Second, while much of the research focuses on satisfaction, study outcomes, and technical capabilities, it largely neglects key areas like regulatory issues, labor market implications, and comprehensive emotional responses associated with ChatGPT use, all of which are essential to fully understanding the integration of AI in educational settings. Third, there is limited analysis of how socio-demographic factors, such as field of study, income region, and other socio-economic characteristics, influence students’ perceptions and experiences with ChatGPT. By considering geographic diversity, a variety of ChatGPT aspects, and different student characteristics, this study seeks to fill these gaps by offering a broader view of students’ perceptions of ChatGPT to provide insights that support meaningful integration and enrich learning experiences while preparing students for future challenges.

## Materials and methods

### Study participants and procedure

Our global study targeted higher education students who are currently enrolled at any level in a higher education institution, are at least 18 years old, and have the legal capacity to provide free and voluntary consent to participate in an anonymous online survey. Survey participants were recruited using a convenience sampling method, which involved promoting the survey in classrooms and through advertisements on university communication systems. This method proved effective due to its practical nature, allowing for easy access to potential participants who were readily available and willing to participate in the survey. The survey was initially designed in English. Due to the lack of systematic evidence on students’ early perceptions of ChatGPT, the content of the questionnaire was developed in collaboration with international partners, focusing on the most important aspects related to ChatGPT. This approach ensured that the questionnaire addressed diverse perspectives and captured a comprehensive view of students’ perceptions of ChatGPT in the context of higher education. The preliminary version of the questionnaire was validated with students from Slovenia (see Aristovnik et al. [11]), and the final version was refined based on feedback from pilot testing, enhancing its reliability and relevance for the target population. In order to ensure and achieve a global reach, the questionnaire was later translated into six additional languages, including Italian, Spanish, Turkish, Japanese, Arabic, and Hebrew, by native speakers proficient in English.

The survey was developed using the web application 1KA (One Click Survey; https://www.1ka.si/d/en), which complies with the General Data Protection Regulation (GDPR), ensuring informed consent, anonymity, and confidentiality for all participants. The survey was published online on 9 October 2023 as a prerequisite for submitting requests for ethical procedures to relevant ethics committees/Institutional Review Boards. All participating international partners adhered to local regulations and guidelines regarding ethical approvals. The survey was conducted at varying time intervals by international partners, depending on when ethical approval was obtained in their respective institutions or countries. In cases where ethical approval was not required, the survey followed the first approval granted on 24 October 2023. The survey remained open for data collection until 29 February 2024. This coordinated approach ensured that the survey maintained ethical integrity across all participating regions. Given that ChatGPT was introduced to the general public in November 2022, the survey thus captured early student experiences with a conversational AI chatbot.

By the end of February, 23,218 students from 109 different countries and territories had participated in the survey. Since participants were not required to complete the entire questionnaire, the number of responses varied across questions. The participation was unequally distributed across different countries and territories as follows: 1) over 1,000 responses were collected in each of 4 countries (Ecuador, Spain, Mexico, and Italy); 2) between 500 and 1,000 responses were gathered in each of 9 countries (Romania, Egypt, Tanzania, Ghana, Chile, Palestinian State, Turkey, Cyprus, and Latvia); 3) between 200 and 500 responses were collected in each of 24 countries; and 4) fewer than 200 responses were collected in each of 72 countries. In order to capture the specifics between countries with similar economic development patterns, the participants were grouped into four income regions: low income, lower middle income, upper middle income, and high income, based on the World Bank classification of countries [96].

The sample was dominated by female students, with a considerable majority being first-level students. Most students were studying social sciences and applied sciences, while fewer were studying natural and life sciences, and arts and humanities. A large share of students was engaged either in traditional learning or blended learning. The majority of students resided in urban areas and came from average economic backgrounds. While students from high income and upper middle income regions prevailed, those from lower middle income regions were less represented, with students from low income regions being represented the least. A detailed overview of the socio-demographic and geographic characteristics of the survey participants is presented in Table 1.

**Table 1:** Socio-demographic and geographic characteristics of the survey participants.

Socio-demographic and geographic characteristics	Number	Share (%)
Gender		
Male	9,346	41.2
Female	13,365	58.8
Level of study		
First	18,935	83.4
Second	2,867	12.6
Third	912	4.0
Field of study		
Arts and humanities	2,740	12.1
Social sciences	9,356	41.4
Applied sciences	7,809	34.5
Natural and life sciences	2,717	12.0
Mode of study		
Traditional learning	10,754	47.3
Online learning	2,159	9.5
Blended learning	9,833	43.2
Area of living		
Urban	11,404	64.3
Suburban	3,513	19.8
Rural	2,823	15.9
Economic status		
Significantly below average	1,179	6.6
Below average	3,504	19.7
Average	9,910	55.8
Above average	2,749	15.5
Significantly above average	424	2.4
Income region		
High income	9,391	41.1
Upper middle income	8,090	35.4
Lower middle income	5,223	22.9
Low income	140	0.6

### Measures

The data were collected through an online questionnaire consisting of 42 primarily closed-ended questions aimed at capturing students’ perspectives on their early experiences with ChatGPT. The questionnaire was structured in the form of 11 sections. In addition to socio-demographic characteristics (12 questions from Q1 to Q12), the questionnaire covered several aspects relevant to ChatGPT, including usage (6 questions from Q13 to Q18), capabilities (1 question, Q19), regulation and ethical concerns (4 questions from Q20 to Q23), satisfaction and attitude (2 questions, Q24 and Q25), study issues and outcomes (2 questions, Q26 and Q27), skills development (2 questions, Q28 and Q29), labor market and skills mismatch (2 questions, Q30 and Q31) and emotions (1 question, Q32). Moreover, the questionnaire also covered general study and personal information (8 questions from Q33 to Q40), including additional socio-demographic elements not directly related to ChatGPT, while the last question was about general reflections on ChatGPT (1 question, Q41). Finally, participants were given the option to agree to receive the results of the survey (Q42). However, since the questionnaire required participants to have prior experience with ChatGPT, it was offered in full only to those who had used ChatGPT. Participants who had not used ChatGPT were offered only questions about socio-demographic characteristics, additional study and personal information, and the option to agree to receive the results of the survey. Except for single-choice and open-ended questions, individual statements within a question asking about frequency and agreement were measured on a 5-point Likert scale from 1 (strongly disagree / never) to 5 (strongly agree / always) [97]. A full version of the questionnaire and the dataset are available in the Mendeley Data repository (see Ravšelj et al. [98]).

### Statistical analysis

Data preparation, including merging and cleaning, was conducted using the Python programming language, specifically with the Pandas and NumPy libraries [99]. Data was analyzed with several statistical approaches. Using the mentioned Python libraries, descriptive statistics, including sample description, top two box scores (i.e., the percentage of students who answered with the highest two responses on the 5-point Likert scale), and mean values of student responses were calculated. Moreover, the written responses from students (Q41) were compiled into a word cloud, which was created using the Python Wordcloud Library [100]. To further examine the mean differences between students with varying socio-demographic and geographic characteristics across different aspects, statistical tests, including independent samples t-test and analysis of variance (ANOVA), were performed using the Python Library SciPy [101].

Finally, to analyze the factors influencing the specific usage of ChatGPT among students, an ordinal logistic regression analysis was conducted. This methodological approach was chosen because it is well-suited for examining ordinal outcomes, such as the frequency of different ChatGPT usage. Consequently, it allows us to effectively understand how various factors influence these outcomes across ordered categories, making it the most appropriate technique for analyzing the ordinal dependent variables related to specific ChatGPT usage (Q18e, Q18g, Q18a). The standard interpretation of the ordinal logit coefficient is that for every one-unit increase in the independent variable, the dependent variable is expected to change by its corresponding regression coefficient on the ordinal log-odds scale, assuming the other variables in the model remain constant. In other words, a positive coefficient indicates that students with higher scores on the independent variable are more likely to fall into a higher category. Conversely, a negative coefficient indicates that students with lower scores on the independent variable are more likely to fall into a lower category [102]. Moreover, several independent variables were included in the ordinal regression analysis, covering selected ChatGPT-related factors across different aspects (capabilities (Q19g, Q19f), regulation and ethical concerns (Q23d, Q21c), satisfaction and attitude (Q25b, Q25d), study issues and outcomes (Q26a, Q26b), skills development (Q29i, Q28i), labor market and skills mismatch (Q30e, Q30i), and emotions (Q32l, Q32e)), with socio-demographic and geographic characteristics (presented in Table 1) included as control variables. While the main independent variables of interest were measured on a 5-point Likert scale, most of the control variables were nominal, meaning they were categorical with no inherent order. Therefore, dummy coding was employed to recode these categorical predictors, enabling the regression coefficients of the newly created dummy variables to meaningfully identify between-group differences [103]. The ordinal regression analysis, along with proportional odds and multicollinearity testing (Spearman correlation and multicollinearity diagnostics, including variance inflation factor (VIF) and tolerance (TOL)), was performed using SPSS 28.0 [104].

As already mentioned, since participants were not required to complete the entire questionnaire, the number of responses varied across questions. Accordingly, a complete case analysis approach was applied to address issues with missing data [105]. Assuming "missing completely at random," meaning that the complete cases are a random sample of the originally identified set of cases, this approach is the most common method for handling missing data in many fields of research, including educational research, with statistical packages such as SPSS being the default method supported for a large number of statistical procedures [106].

### Ethical considerations

All participants in the global ChatGPT student survey were provided with detailed information about the study. Participation was anonymous and voluntary, with students having the option to withdraw at any time without any consequences. To ensure data protection, the online survey was only available to individuals aged 18 or older who were enrolled in a higher education institution. Before starting the survey, the participants were required to provide a written agreement to the terms of participation by clicking ’Next page’ on the introductory page of the online questionnaire, thereby consenting to the outlined conditions and agreeing to participate in the survey. This consent procedure was reviewed and approved by the relevant ethics committees/Institutional Review Boards as part of the ethical review process, ensuring that it meets the necessary ethical standards for participant consent. The procedures of this study complied with the provisions of the Declaration of Helsinki for research involving human participants. Ethical committees of several involved higher education institutions approved this study, including the University of Oran 1, Algeria (Ethical Clearance Number: 03/CED/FACMED/2023); the University of Nicosia and the European University Cyprus, Cyprus (Ethical Clearance Number: EEBK EII 2023.01.318); the Polytechnic University, Ecuador (Ethical Clearance Number: C-22); the University of Verona, Italy (Ethical Clearance Number: 2023_25); Yamanashi Gakuin University, Japan (Ethical Clearance Number: 23–010); the University of Luxembourg, Luxembourg (Ethical Clearance Number: ERP 23–101 StuPer ChatGPT SA/cd); Imam Abdulrahman Bin Faisal University, Saudi Arabia (Ethical Clearance Numbers: IRB-2024-02-091 and IRB-2024-10-316); the University of Chester, United Kingdom (Ethical Clearance Number: ASCHPR0211/23); and the University of East London, United Kingdom (Ethical Clearance Number: ETH2324-0028). Additional information regarding the ethical, cultural, and scientific considerations specific to inclusivity in global research is included in the Supporting Information (S1 Checklist).

## Results

A first insight into early student experiences with ChatGPT revealed that most students (71%) had already used ChatGPT, providing valuable insights into their early experiences with the tool during its first year of existence. While only the students who have used ChatGPT are further elaborated on, a profile of the students who have not used ChatGPT can still be extracted, revealing the characteristics of this subgroup. According to the socio-demographic and geographic characteristics of those who have not used ChatGPT, the majority were female (68%), first-level students (84%), students studying social sciences (46%), engaged in traditional learning (52%), residing in urban areas (60%), from average economic backgrounds (58%), and studying in high income (39%) and upper middle income (34%) regions.

### Overview of the survey results

**Fig 1:**
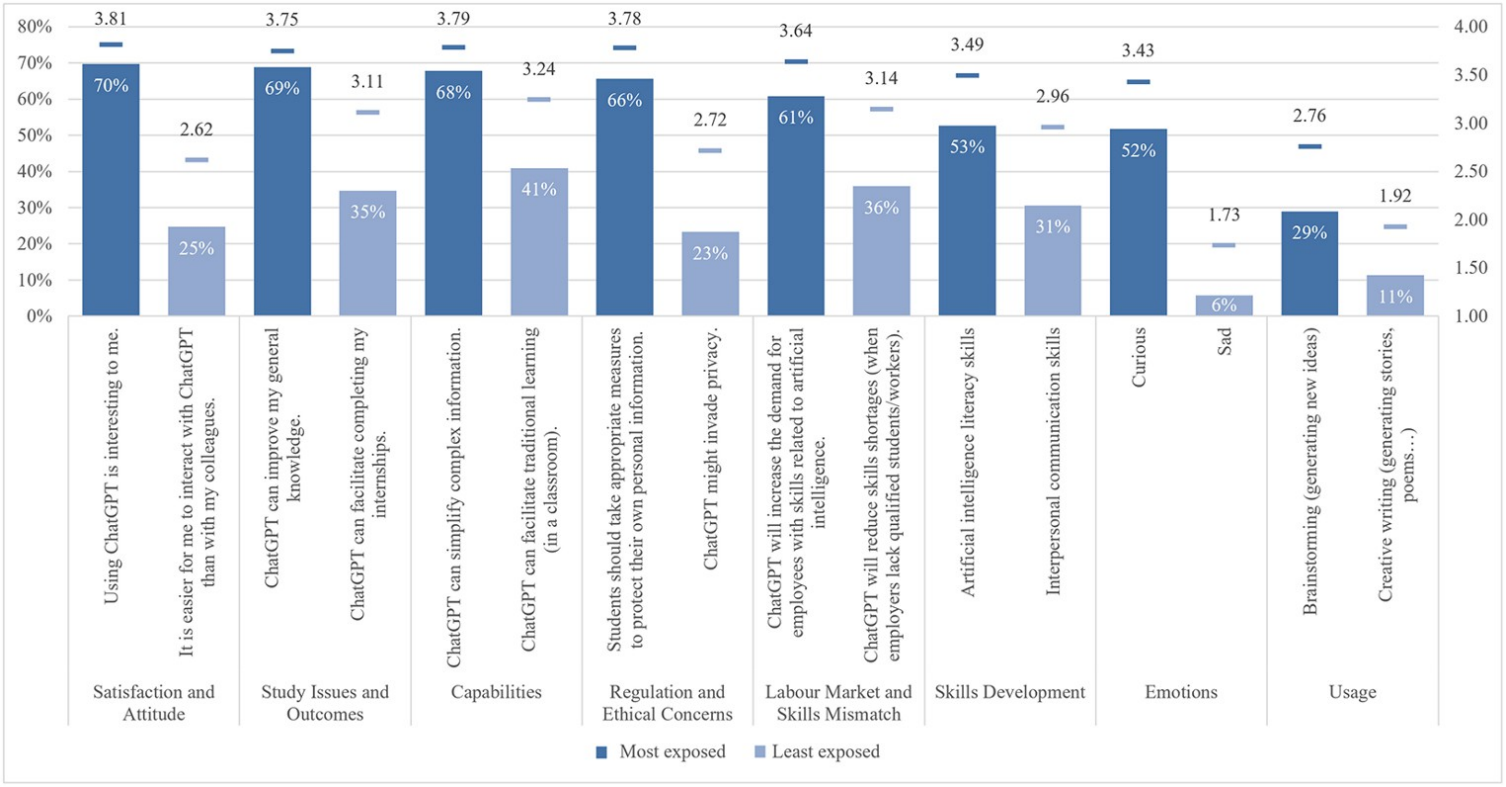
Most and least exposed statements across ChatGPT aspects.

The global survey results include student perceptions of ChatGPT, highlighting the most and least emphasized elements (statements) across various aspects (Fig 1). In terms of satisfaction and attitude, most students (70%) found ChatGPT interesting to use, while only a quarter found it easier to interact with ChatGPT than with colleagues. For study issues and outcomes, the majority of students (69%) reported that ChatGPT can improve their general knowledge, whereas only about one-third indicated it can facilitate completing their internships. Regarding capabilities, most students (68%) valued its ability to simplify complex information, whereas 41% noted its support for traditional classroom learning. Under regulation and ethical concerns, most students (66%) were aware of taking appropriate measures to protect personal information, compared to slightly less than one-quarter who were concerned about privacy invasion. In the labor market and skills mismatch aspect, most students (61%) saw that ChatGPT would increase the demand for employees with AI-related skills, while fewer (36%) acknowledged its potential to reduce skills shortages. For skills development, about half of the students (53%) perceived ChatGPT as an effective tool to improve their AI literacy skills, while less than one-third (31%) believed it was effective in enhancing their interpersonal communication skills. Emotions-wise, about half of the students felt curious using ChatGPT, while only 6% felt sad. Lastly, regarding usage, less than a third of students (29%) used it for brainstorming, and only a few (11%) for creative writing.

**Fig 2:**
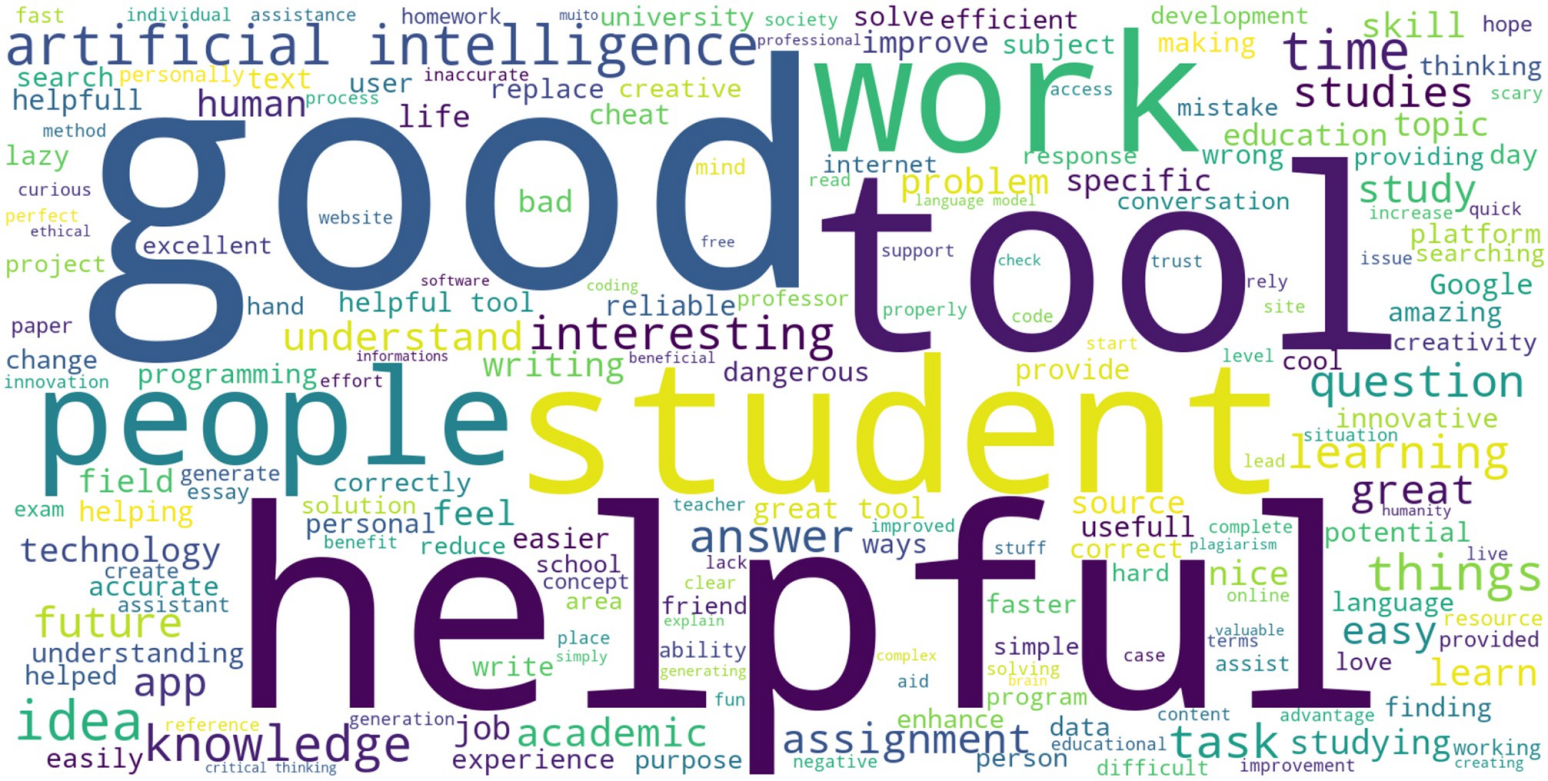
Word cloud of students’ perceptions of ChatGPT.

Moreover, general views about students’ perceptions of ChatGPT were gathered from students’ written responses by asking them to write down their views on how they see ChatGPT. As illustrated in the word cloud visualization (Fig 2), the results highlight several key themes and insights relevant to ChatGPT’s application and perception among students. The most prominent words are "good," "helpful," "tool," and "student," indicating that students view ChatGPT as a valuable tool for their activities. The emphasis on "work," "task," "assignment," and "studies" shows its usefulness in managing coursework. Key themes like "learning," "knowledge," and "understand" highlight its role in enhancing comprehension. Frequent mentions of "question" and "answer" suggest its utility in providing quick responses, while words like "idea," "thinking," and "interesting" indicate its ability to stimulate creativity. Positive terms such as "great," "improve," "amazing," and "future" underscore its value and potential in education. Words like "technology," "AI," "innovation," and "app" reflect recognition of ChatGPT as a cutting-edge tool. Terms like "easy," "efficient," "fast," and "reliable" show it is user-friendly and dependable. Words like "academic," "university," "professor," and "reference" suggest it is seen as a credible aid in higher education. Negative terms like "cheat," "plagiarism," and "lazy" indicate some ethical concerns, but these are less prominent. Overall, the word cloud illustrates that students view ChatGPT as a beneficial, innovative tool that enhances their academic experience and holds promise for the future of education.

The supporting information (S1 File) includes a comprehensive table with detailed empirical results for each aspect of ChatGPT. The table is organized by different aspects, clearly indicating which statement pertains to each aspect along with the mean and top two box values for the total sample for each statement, allowing for the identification of the most and least exposed statements across aspects by both criteria. Moreover, the table presents comparisons between different students’ groups based on diverse socio-demographic and geographic characteristics. Specifically, the comparison includes: 1) information about the highest and lowest mean values achieved in specific groups of students; 2) mean differences between the highest and lowest mean values, supplemented by the significance of these differences; 3) information on which group of students (based on socio-demographic and geographic characteristics) reported the highest and lowest mean values; and 4) information on which group of students (based on socio-demographic and geographic characteristics) reported the highest and lowest top two box scores. The results of the comprehensive analysis are systematically presented in the following subsections.

#### Usage

**Fig 3:**
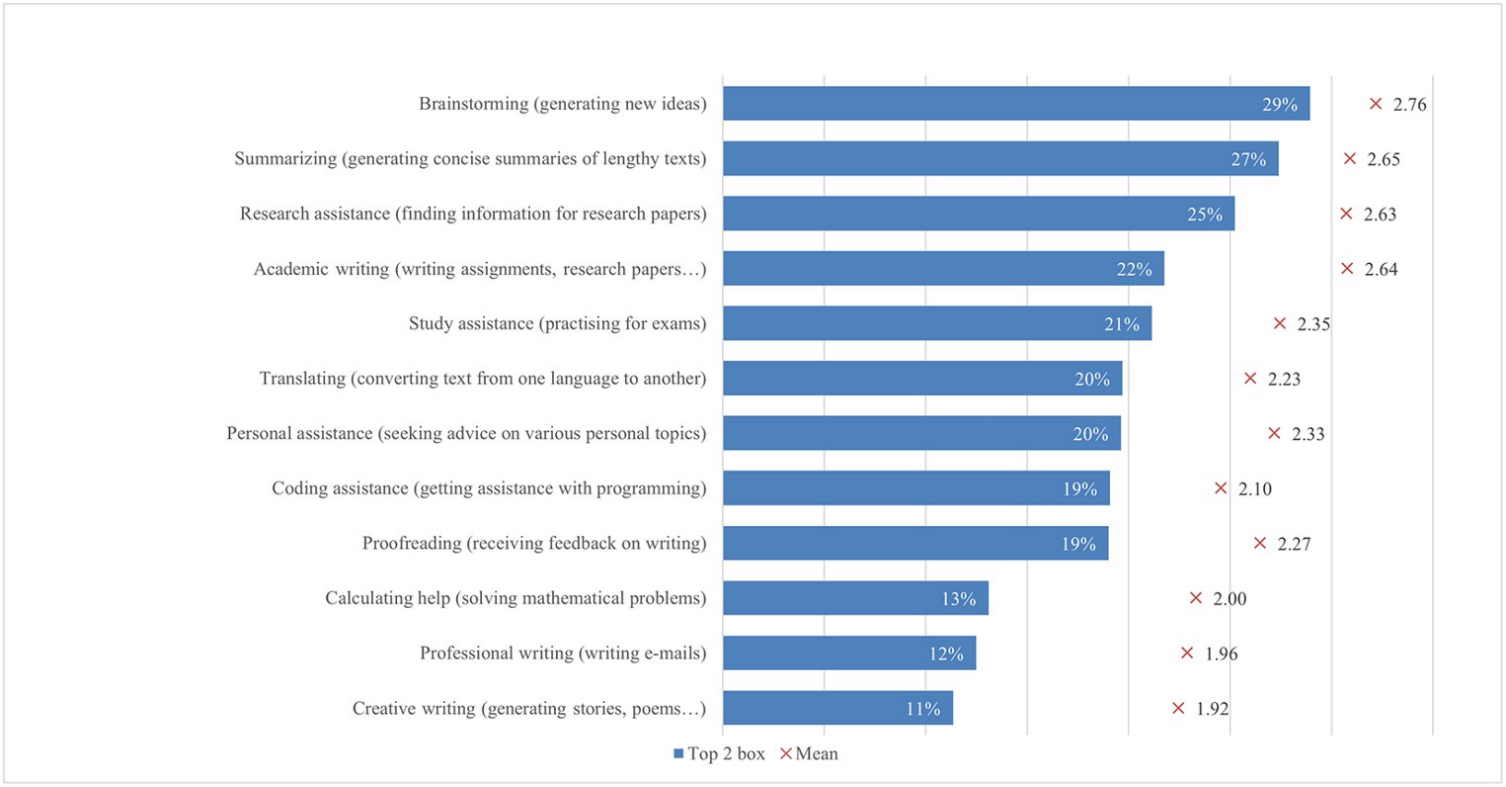
ChatGPT usage frequency (top two box scores and average values).

The possibilities and uses of ChatGPT offer a wide range of options to the students in their learning process. However, how students use the opportunities offered by the new technology varies according to their abilities, interests, affection and integrity (e.g., Bouteraa et al. [23], Garrel & Mayer [107], Shoufan [85]). In the first section of the questionnaire, students were asked how often they used ChatGPT for different tasks, from more general tasks such as proofreading, translating, or asking for advice on different topics to more specific tasks such as help with academic writing or coding (Fig 3). On average, students’ responses ranged from 1.92 to 2.76. Students most frequently reported using ChatGPT for brainstorming, with almost 30% (2.76), followed by using ChatGPT for summarizing long texts, which was supported by 27% of students (2.64), finding articles for research, supported by 25% of students (2.63), and writing texts, chosen by 22% of students (2.64), which is partially consistent with Chan and Hu’s [30] findings. The lowest number of students used ChatGPT for professional writing, supported by 12% of students (1.96), and creative writing, chosen by 11% of students (1.92). As both these writings are very personal in nature, this could mean that students still prefer to express themselves personally rather than leave it to the AI.

**Fig 4:**
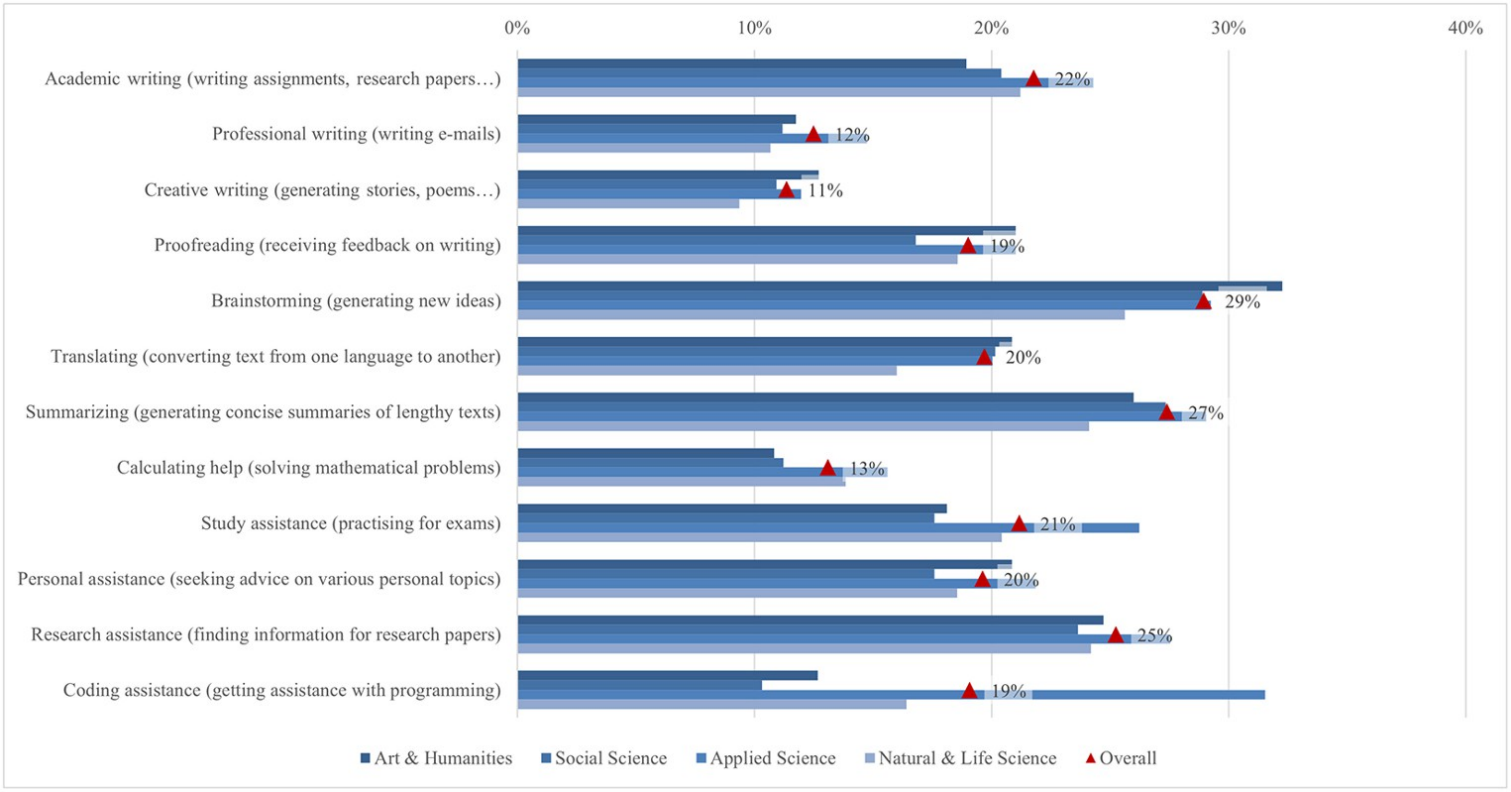
Differences in ChatGPT usage frequency (top two box scores by field of study).

Unlike Garrel and Mayer [107], who found that engineering, mathematics, and science students used ChatGPT most frequently, the responses in our survey showed that the frequency of use differed between disciplines on average from 1.75 and 2.88 (Fig 4). Arts and humanities students were more likely to use it for creative writing, proofreading, brainstorming, translation, and personal support, while applied sciences students were more likely to use it for academic and professional writing, summarising, calculating and coding, as well as for study and research support. Focusing on the student’s field of study, the largest difference (0.84) between the highest and lowest mean was found in coding support for programming, which is not surprising since the difference observed is between students of applied sciences with almost 32% engagement, where programming is more often a compulsory subject, and those of social science, where students are not often interested in coding computer programs (10%). In fact, this is where the largest differences were found in the whole questionnaire. Coding assistance also showed one of the larger gender differences (0.58), with males scoring higher. As there are more men than women studying programming or related computer studies, this is not surprising. The use of ChatGPT for generating new ideas and brainstorming is the most popular among students, varying between 25% and 33% depending on the field of study. In fact, a third of arts and humanities students have used it, but the average usage was still low (2.88). When students were looking for information to write a research paper, the smallest difference was found between the fields (0.11), with almost a quarter of students using ChatGPT, regardless of their field of study.

**Fig 5:**
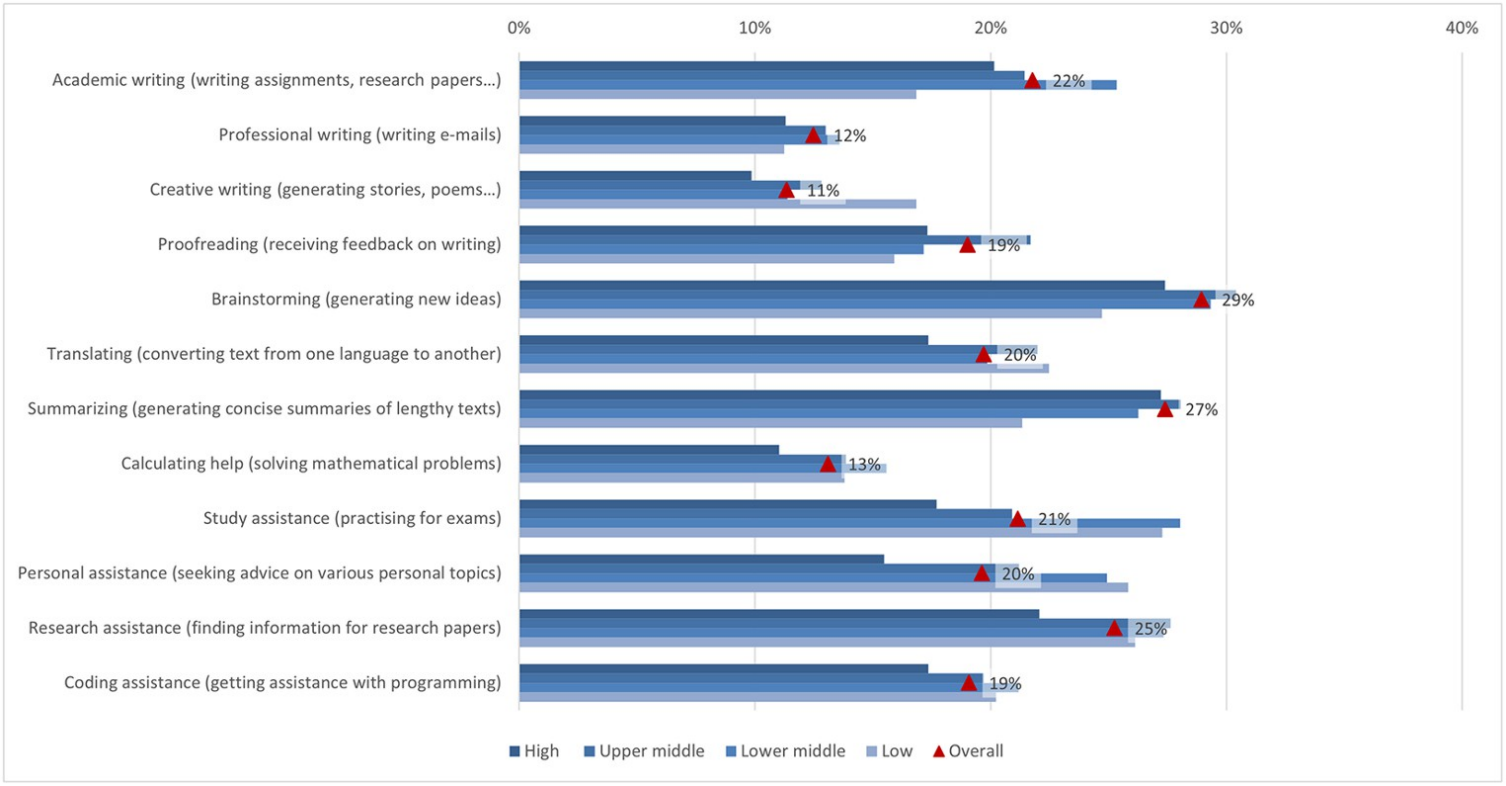
Differences in ChatGPT usage frequency (top two box scores by income region).

In general, according to the income region, there was a notable gap between the demographic groups (Fig 5). Interestingly, in almost all cases, the highest scores were achieved by students from low and lower middle income regions, while those from high income regions scored lowest on all statements. Hence, the largest difference was found for using ChatGPT to help with learning (0.64), followed by personal help with various tasks (0.54), for using ChatGPT to help with coding (0.51) and academic writing (0.41). Overall, we can summarize that the tasks for which students used ChatGPT, supported by 25% or more of the students regardless of income region, were generating new ideas, summarizing long texts concisely, and helping with research writing.

However, if we look at the results according to different demographic factors, the results show that significantly lower scores for usage were achieved by males, in traditional education, by students with a lower than average economic status and also by students from the low income regions. If, in the first two, it is still possible to speculate about the personal approach, then the last two demographic groups may indicate that this type of writing is not often used by students from economically disadvantaged backgrounds. It is also notable that students from rural backgrounds demonstrated the highest usage of ChatGPT, regardless of the task. However, the differences between demographic groups based on their place of residence were minimal. This pattern is also observed for students with significantly abovenaverage economic status, where the differences were more pronounced. Additionally, there were no major differences between the groups when considering the level of study, except for the use of ChatGPT as a study assistant, reported by 22% of students in the first level of study. This might be because students at higher levels encountered ChatGPT later in their academic journey, by which time they had already developed their own learning strategies.

#### Capabilities

**Fig 6:**
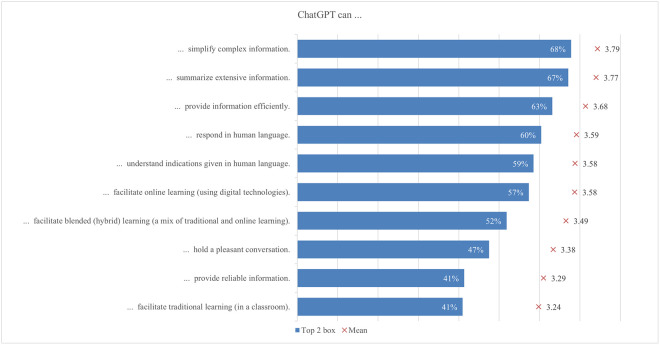
Agreement on ChatGPT capabilities (top two box scores and average values).

Students were generally aware of ChatGPT’s capabilities, particularly in simplifying complex information (68%) and summarizing extensive content (67%) (Fig 6). This awareness highlights students’ recognition of ChatGPT as a valuable tool for breaking down difficult concepts and condensing large amounts of information into more manageable forms. However, respondents were least likely to believe that ChatGPT could provide reliable information (41%) and support traditional classroom learning (41%). This discrepancy implies that while students appreciate the strengths of ChatGPT in making information easier to understand and summarizing content, they remain skeptical about its reliability and usefulness in a traditional classroom setting. This skepticism is also reflected in the findings of Mai et al. [108], who noted similar concerns about the reliability and classroom integration of AI tools like ChatGPT.

**Fig 7:**
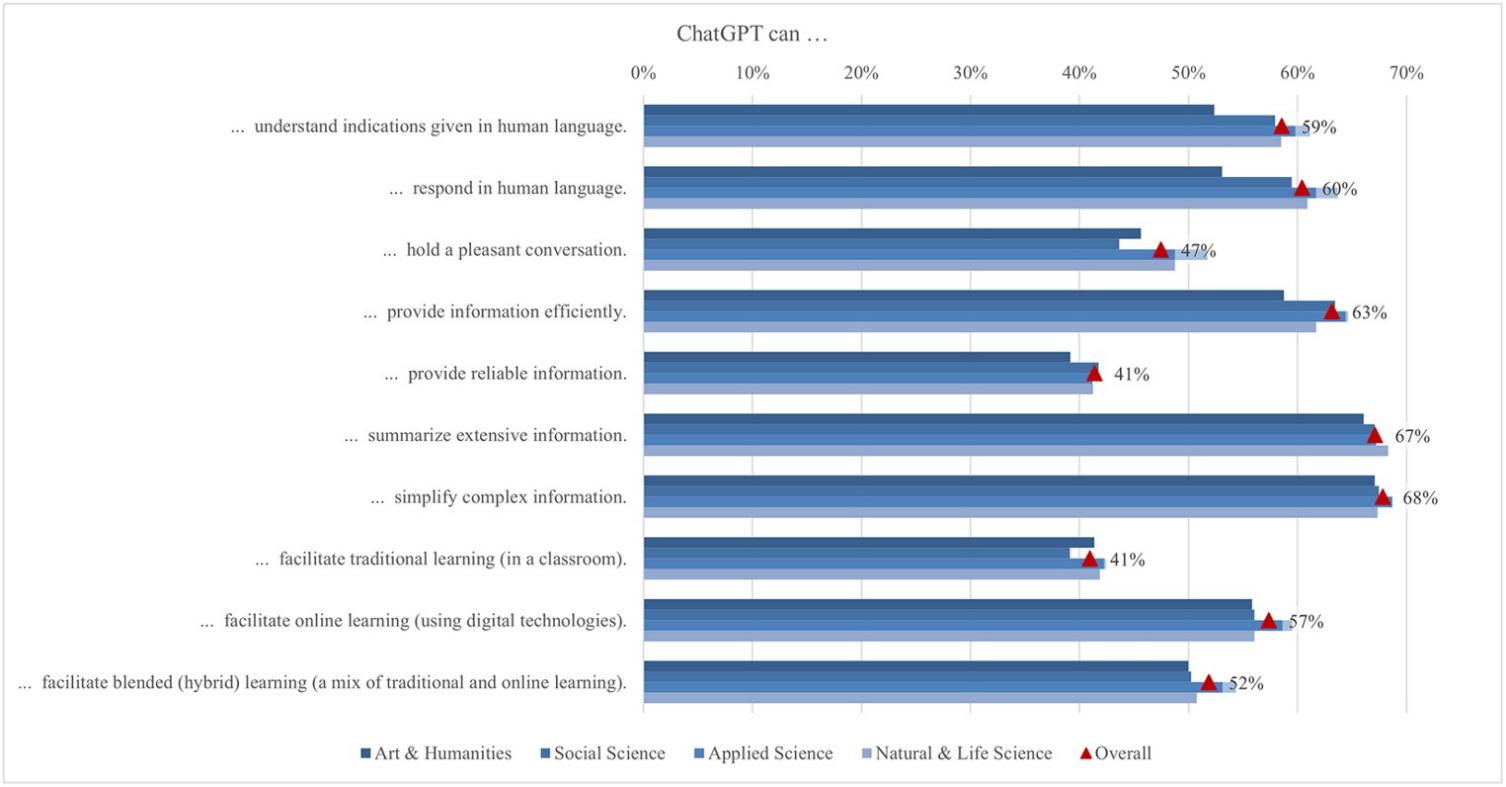
Differences in agreement on ChatGPT capabilities (top two box scores by field of study).

To some extent, academic discipline seems to influence perceptions of ChatGPT’s capabilities, particularly its ability to respond in human language (Fig 7). Despite lacking feelings, unique experiences, or subjective viewpoints, ChatGPT is designed to simulate human-like interaction and respond naturally, creating the perception of a human touch [28]. Applied sciences students (64%) found this ability more beneficial, as ChatGPT’s capability in simplifying and clarifying complex technical information aligns with their field’s emphasis on clear and accurate communication. In contrast, arts and humanities students (53%), who prioritize nuanced and interpretive language, might be more critical of ChatGPT’s ability to capture the depth and subtleties of human expression. This indicates that while ChatGPT is valued for its technical prowess in certain disciplines, its perceived shortcomings in handling more abstract and subjective content limit its acceptance among students in fields that demand such capabilities.

**Fig 8:**
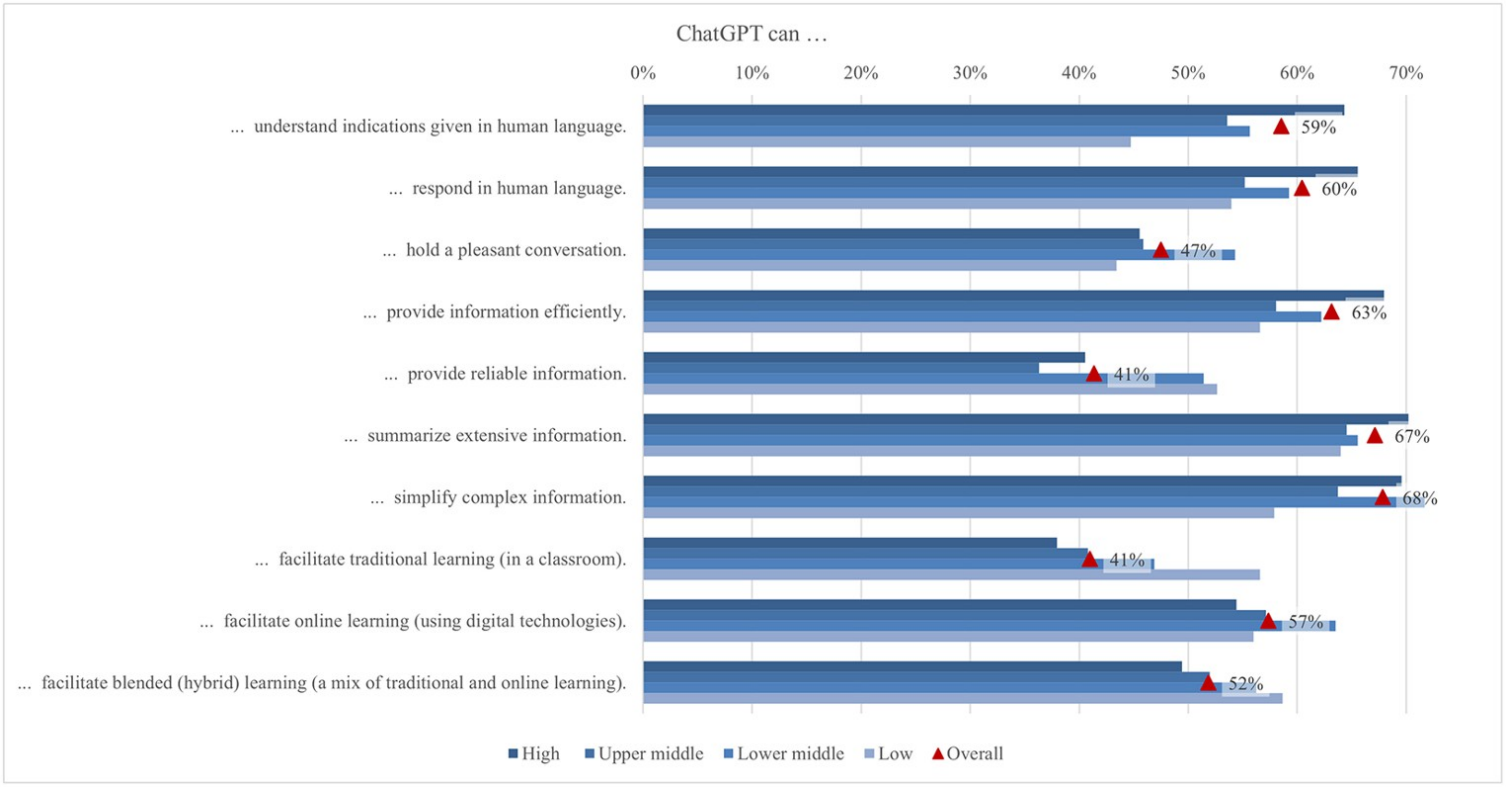
Differences in agreement on ChatGPT capabilities (top two box scores by income region).

Larger gaps in perceiving ChatGPT’s capabilities were observed across income regions, with students from low income regions (57%) being the most aware of ChatGPT’s potential to support traditional classroom learning, while students from high income regions are the least aware (38%), confirmed by the largest mean difference (0.44) (Fig 8). This could be due to the greater reliance of students from low income regions on cost-effective digital tools to supplement their lack of educational resources [109]. Conversely, students from high income regions (64%) seem to be the most aware of ChatGPT’s capability to understand instructions given in human language, while students from low income regions (45%) perceived it the least. This finding aligns with the observation that developed countries have already implemented modern learning approaches emphasizing personalized learning, making ChatGPT’s capability to understand human language more appreciated by students from developed countries compared to those from developing countries [110]. Thus, the economic context significantly shapes how students perceive and utilize AI tools in their educational pursuits.

While gender and area of living had only minor implications for student perceptions of ChatGPT’s capabilities, more noticeable differences could be observed in other socio-demographic characteristics. Compared to others, students of above average economic status largely agreed that ChatGPT can understand instructions given in human language (66%). This agreement likely stems from their exposure to and familiarity with advanced technological tools and educational resources. Additionally, blended learning students largely agreed that ChatGPT could facilitate blended learning (58%), highlighting the tool’s potential to support hybrid educational models that combine online and face-to-face instruction. Finally, despite relatively lower percentages across levels of study, undergraduate students largely agreed that ChatGPT could provide reliable information (42%). This indicates a cautious optimism among undergraduates about the potential of AI tools to enhance their learning experience despite prevailing concerns about their reliability.

#### Regulation and ethical concerns

**Fig 9:**
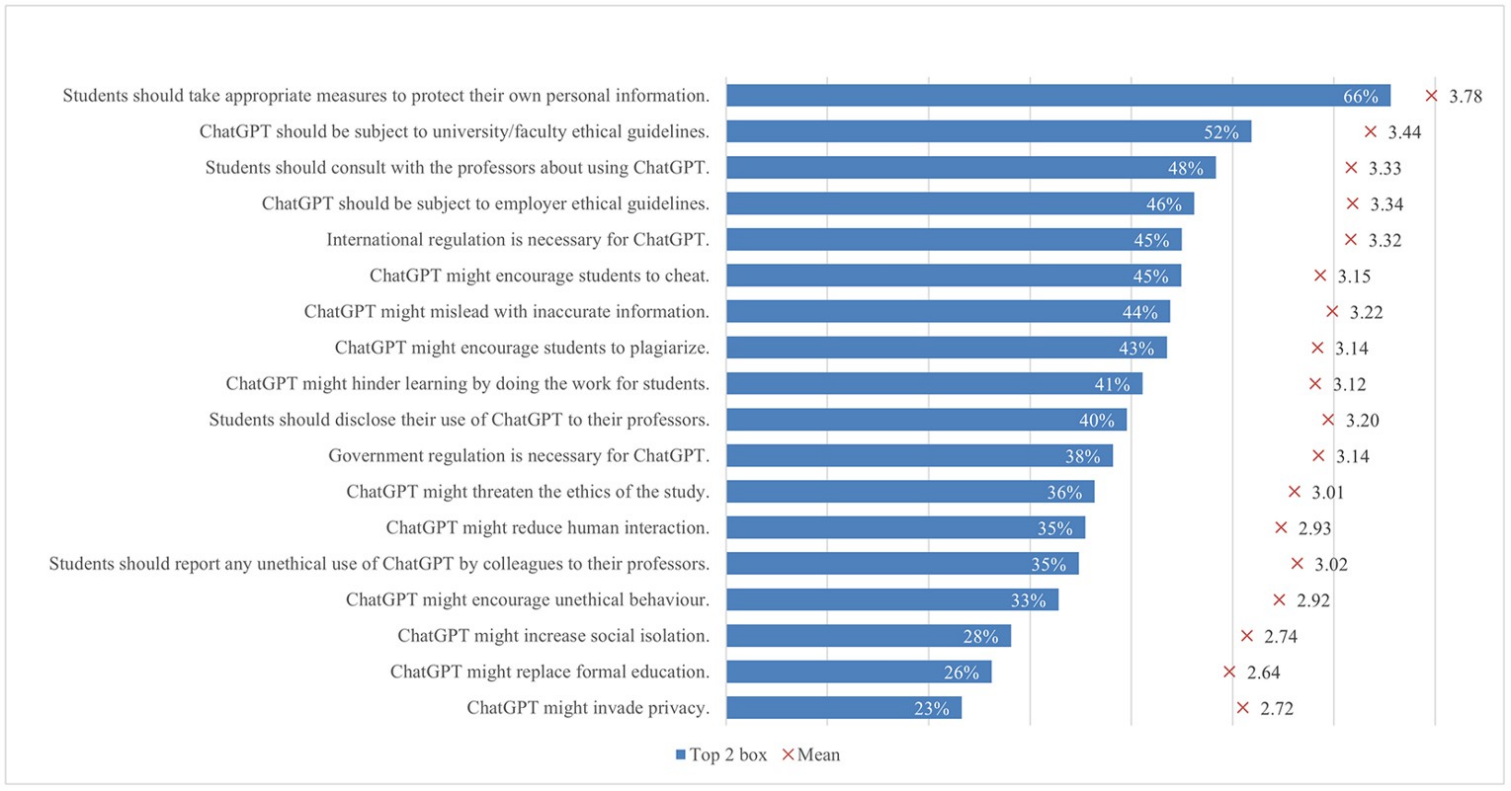
Agreement on ChatGPT regulation and ethical concerns (top two box scores and average values).

There is a consensus in academia about different ways of regulating AI usage, as proposed by several studies [111, 112]. The results of our study also reflected this consensus among students regarding the need for international and government regulation of AI systems like ChatGPT, with mean values of 3.32 and 3.14 (Fig 9). Particularly relevant to students, there was a strong agreement on the necessity of ethical guidelines from universities, faculties, or employers, as indicated by mean scores of 3.44 and 3.34. This agreement on ethical guidelines aligned closely with the students’ awareness of potential issues associated with ChatGPT. They were concerned that ChatGPT could promote unethical behaviors, especially cheating and plagiarism, reflected in mean values of 3.15 and 3.14, respectively. Additionally, there were fears that these systems might compromise the ethics of study and mislead users with inaccurate information. This heightened awareness among students was supported by numerous studies testing the accuracy of information produced by AI [111, 113]. On the other hand, students also expressed concerns about the social impacts of ChatGPT, including privacy invasion, reduced human interaction, and increased social isolation. Overall, the findings underlined the importance of balancing the potential benefits and risks of AI. While AI has the potential to revolutionize industries [114], the necessity of protecting personal information is clear, as emphasized by a considerable share of students (66%).

**Fig 10:**
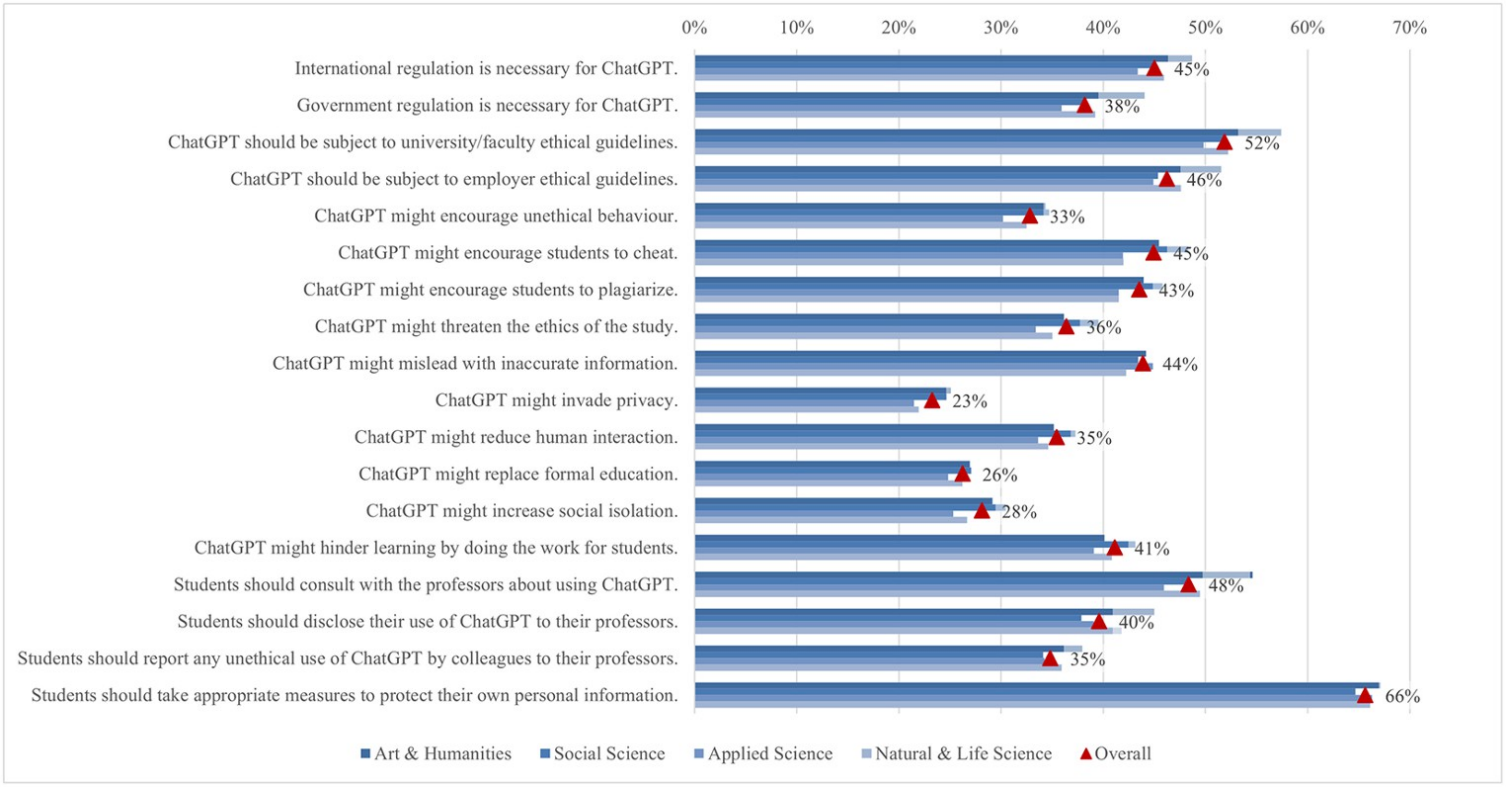
Differences in agreement on ChatGPT regulation and ethical concerns (top two box scores by field of study).

While limited research has been conducted on how regulatory and ethical concerns regarding ChatGPT vary across academic disciplines, our study found that opinions on this matter differ remarkably among these groups (Fig 10). Students from arts and humanities showed the strongest inclination toward regulatory support across all settings, particularly in the belief that ChatGPT should be subject to university and faculty ethical guidelines (57%). Although approximately 50% of students from applied sciences supported regulation in academic settings, their support was consistently the lowest across various contexts, including international, government, academic, and employment regulations. Students from arts and humanities and social sciences generally expressed greater concern about whether ChatGPT encourages cheating, plagiarism, and unethical behavior compared to those from applied sciences and natural and life sciences, as indicated by significant differences ranging between 0.14 and 0.15. Although relatively fewer students were concerned about whether ChatGPT might invade privacy, replace formal education, or increase social isolation, those from arts and humanities and social sciences still demonstrated greater concern compared to students from applied sciences and natural and life sciences. In terms of ethical considerations, a larger proportion of students from arts and humanities believed they should consult (55%) or disclose their usage of ChatGPT to their teachers (45%), which was significantly higher than in all other disciplines. In general, students from applied sciences and natural and life sciences tend to have lower concerns about ethical and regulatory issues regarding ChatGPT usage, which could stem from their comfort with and understanding of technological tools [115–117]. This familiarity with new technologies may lead these students to adjust their ethical attitudes about regulations in a way that aligns with their personal interests, a behavior explained by cognitive dissonance theory, which posits that individuals modify their beliefs to reduce discomfort from conflicting attitudes [118], in contrast to students from arts and humanities, and social sciences who may demonstrate greater caution or skepticism.

**Fig 11:**
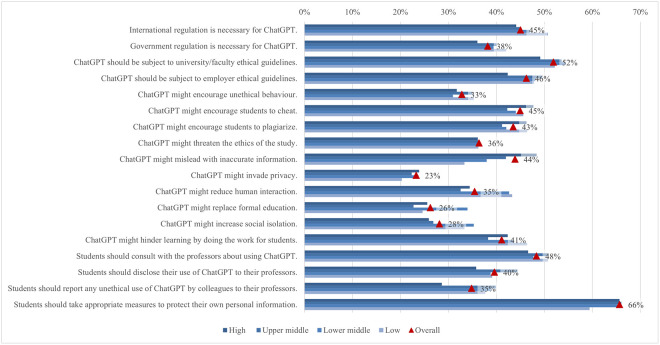
Differences in agreement on ChatGPT regulation and ethical concerns (top two box scores by income region).

Students from high income regions showed significantly less support for official government regulation (36%), university guidelines (49%), and employer guidelines (42%) (Fig 11). Similarly, they were least likely (29%) to report unethical use of ChatGPT by colleagues to their teachers, compared to students from other income regions. An interesting trend observed was that as the income level of a region increases, so does the concern about the potential for misleading information due to AI. High income regions showed the greatest concern (48%), followed by upper middle (42%), lower middle (38%), and low income regions (33%). Additionally, low income regions were notably more worried about social issues such as increased social isolation and reduced human interaction, as indicated by significant mean differences of 0.29 and 0.24, respectively.

A consistently higher percentage of female students compared to male students believed that there should be ethical guidelines and regulations at the international, government, university, and employer levels. They also consistently expressed more ethical concerns about ChatGPT, including issues related to ethics and social impact. The heightened moral concerns among females, observed across different cultures, may be attributed to various socio-cultural factors [119, 120]. Another trend consistently appeared across most statements measuring concern about regulations and ethical issues, indicating that students with more advanced levels of study typically exhibit a heightened awareness of the implications of AI use, which develops over their time in college. Finally, the area of residence seemed to influence concerns, i.e., students living in urban and suburban areas were slightly more concerned that ChatGPT encourages cheating and plagiarism (approximately 45%), compared to about 40% of students from rural areas.

#### Satisfaction and attitude

**Fig 12:**
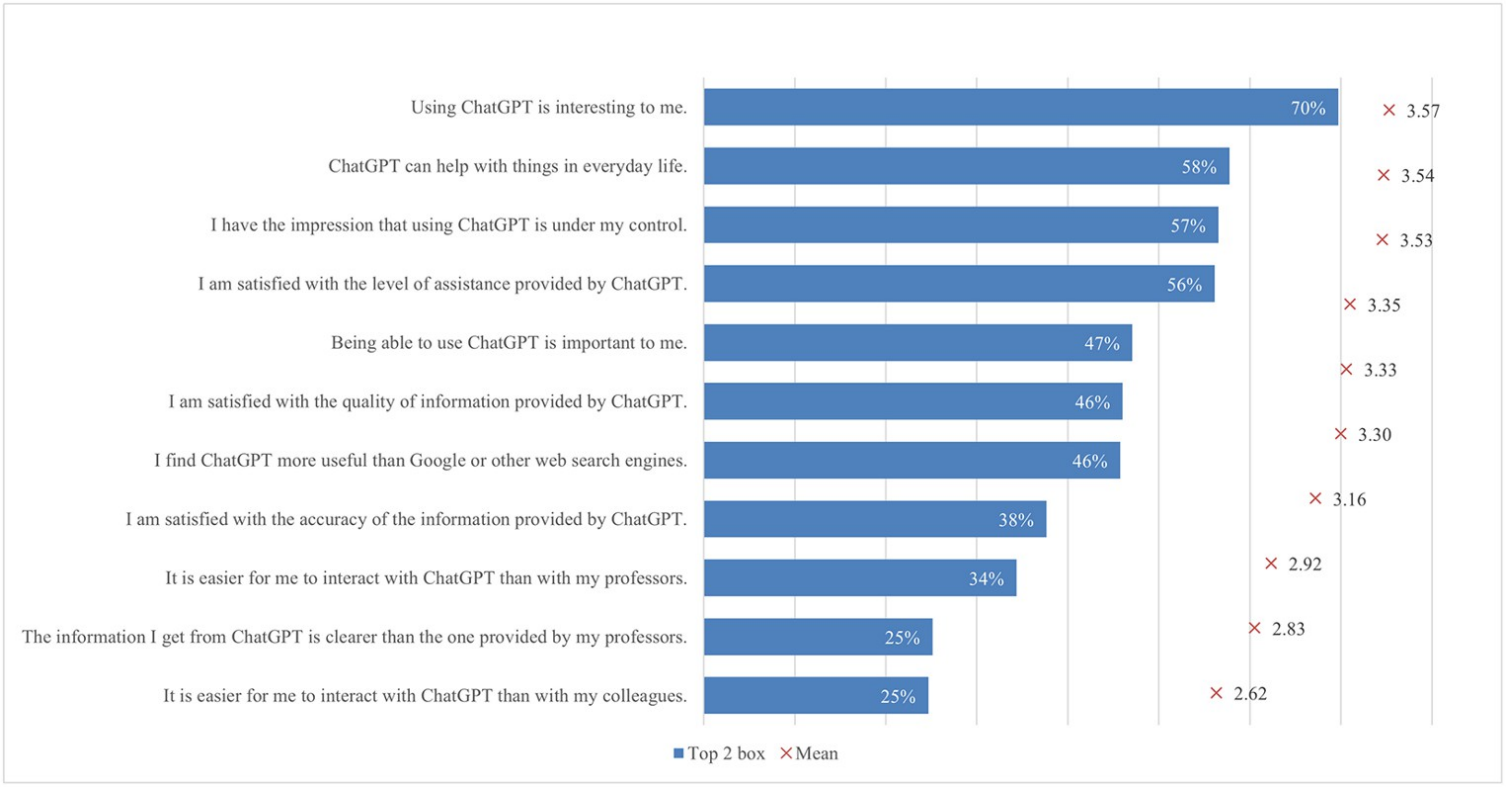
Agreement on ChatGPT satisfaction and attitude (top two box scores and average values).

On average, students’ satisfaction ranged from 2.62 to 3.83. In comparison, the ratings for the attitude statements were somewhat higher, ranging from 3.35 to 3.81 (Fig 12). Almost 70% of students agreed or strongly agreed that using ChatGPT is interesting. A good half of students agreed that ChatGPT is helpful in their daily lives (58%), that they can control it (57%), and that they are satisfied with the level of its assistance (56%). Conversely, only 25% of students agreed or strongly agreed that it is easier to interact with ChatGPT than with colleagues and that the information obtained from ChatGPT is clearer than that provided by their teachers. The preference for human interaction, noted by Rodway and Schepman [121], can be attributed to the lack of social presence in AI interactions [122]. This theory suggests that personal connection and interaction quality are often lower in computer-mediated communication than in face-to-face interactions, making students less comfortable with an AI tool. The low percentage of students agreeing that ChatGPT provides clearer information than their teachers may stem from a "human favoritism" bias rather than an aversion to AI [123, 124]. This bias means content created by humans is rated higher despite AI-generated content sometimes matching or exceeding human quality. Overall, in our study, students were generally satisfied with ChatGPT’s usefulness and assistance but preferred human interaction and communication clarity, highlighting areas for AI improvement.

**Fig 13:**
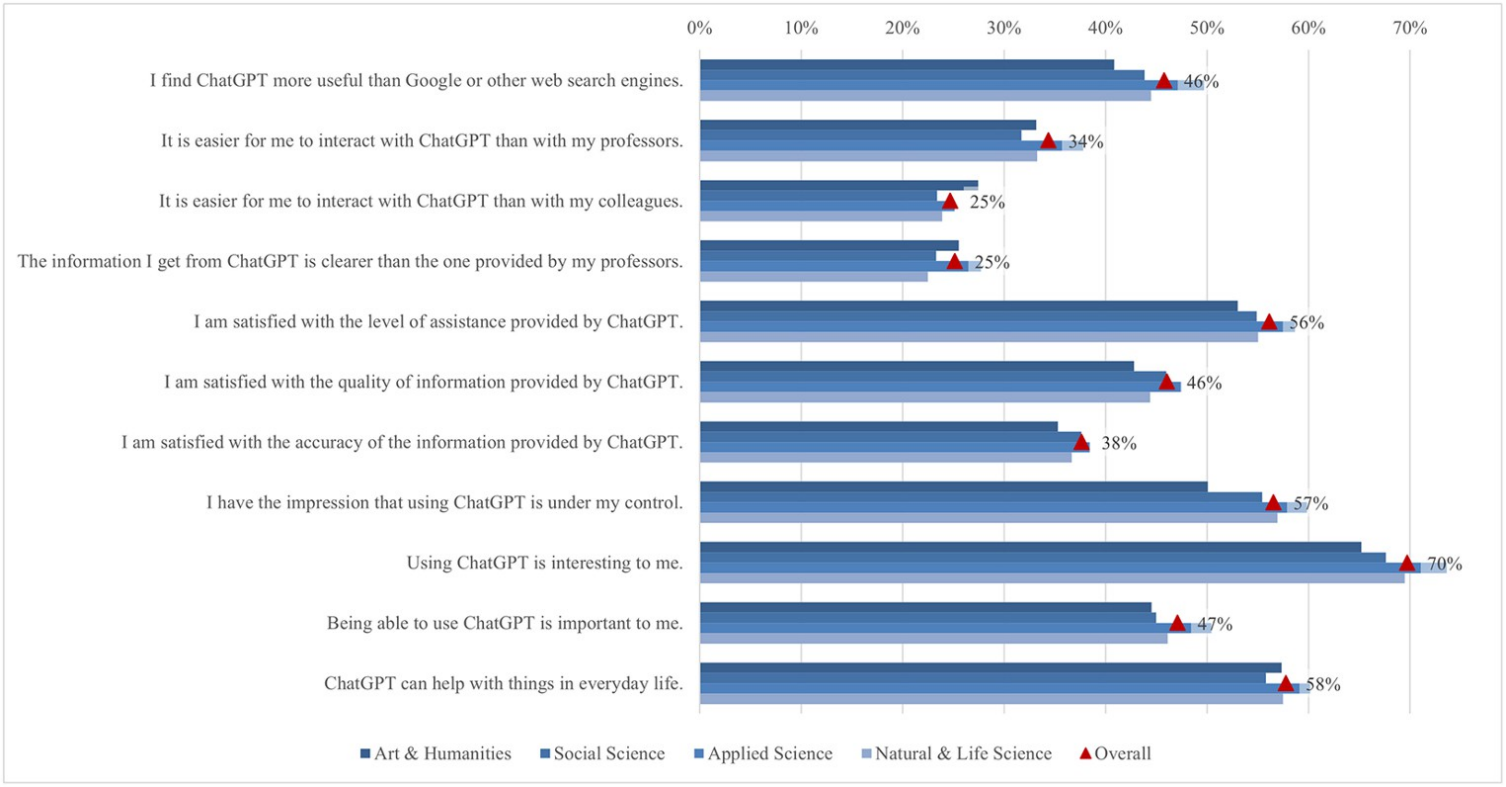
Differences in agreement on ChatGPT satisfaction and attitude (top two box scores by field of study).

Examining the data by field of study reveals significant differences in student satisfaction and attitudes towards ChatGPT (Fig 13). In general, students in applied sciences consistently reported higher levels of satisfaction with all statements compared to their peers in other subject areas but did not deviate from the results regarding the proportions of agreement mentioned above. The differences in satisfaction might be explained by the nature of the disciplines and the inherent demands of students’ academic work. Students in applied sciences often use digital tools and technologies as part of their curriculum, which requires them to interact with software, coding, and data analysis tools. The pedagogical approach in applied sciences emphasizes practical applications and problem-solving, where ChatGPT can be significantly supportive. ChatGPT’s ability to provide clear, factual, and technical support meets these needs, increasing perceived usefulness and satisfaction [125]. Conversely, social sciences and humanities involve subjective analysis, critical reflection, and personal interpretation, areas where human educators excel. Thus, students in these disciplines may show lower satisfaction and more negative attitudes towards ChatGPT.

**Fig 14:**
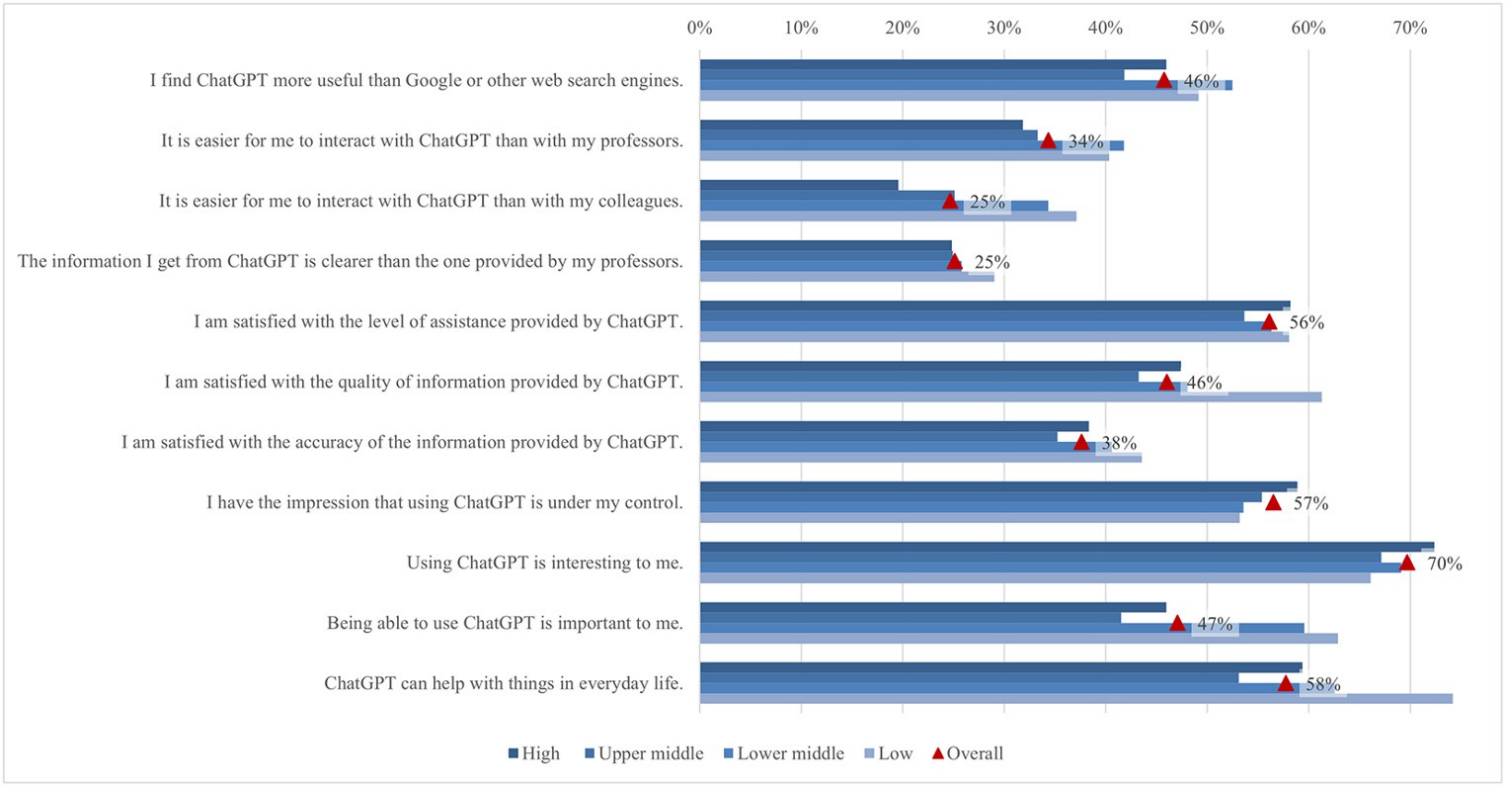
Differences in agreement on satisfaction and attitude (top two box scores by income region).

Additionally, significant rating differences were found between students from different income regions (Fig 14). Students from high income regions were more likely to emphasise their interest in using ChatGPT (72%), with a mean score of 3.85. In contrast, students from low income regions focused more on the benefits of everyday life (74%, 3.87). This highlights a difference in how students from different income regions perceive and use ChatGPT, with high income students valuing its innovative aspects and low income students valuing its practical help. These differences can be attributed to varying levels of access to technology, expectations, and educational support. Moreover, students from low or lower middle income regions rate ChatGPT as the most useful, easy to use, quality, accurate, and important source of information. This may be because it fills critical gaps in their educational resources, which are often limited. On the other hand, students from high income regions feel the most in control, possibly due to their higher familiarity with technology and more robust support systems. The largest difference between the mean maximum and minimum values (0.71) was found in the interaction with ChatGPT compared to their peers, where students from low income regions showed higher agreement, with 37% agreeing or strongly agreeing.

Furthermore, the results highlight significant differences in satisfaction and attitudes towards ChatGPT based on various demographic factors. Male students reported higher levels of satisfaction and more positive attitudes than female students in all statements, echoing findings by Schepman and Rodway [126], who reported more positive AI attitudes in men, which may be due to greater comfort with technology. First level students generally reported higher levels of satisfaction and more positive attitudes regarding ChatGPT than students at later levels. Blended learning students and those living in urban areas find ChatGPT more useful and easier to interact with, probably due to better access to digital resources. Economically disadvantaged students reported high levels of satisfaction with the accuracy and usefulness of ChatGPT, indicating its central role in bridging educational gaps but also the risk of a social/digital divide. These differences highlight the importance of tailoring AI tools to meet the diverse needs of students from different demographic backgrounds. One of the main concerns with using ChatGPT is satisfaction with its accuracy for academic tasks, particularly with complex or context-specific queries and issues related to academic integrity [50]. Results of our study showed that 38% (3.16) of students were satisfied with the accuracy of the information provided by ChatGPT. Satisfaction with the accuracy of ChatGPT was higher among male students and first level students. More positive attitudes towards AI may be associated with male satisfaction. On the other hand, the satisfaction of first level students may be explained by the fact that younger students have less experience with alternative academic tools in contrast to higher level students, who have more experience with academic resources and be more critical of the accuracy of the information they receive.

#### Study issues and outcomes

**Fig 15:**
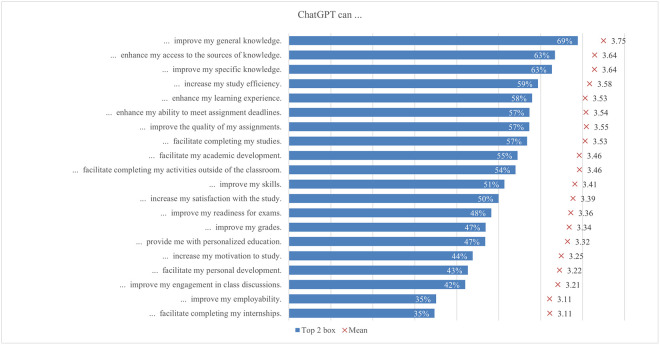
Agreement on study issues and outcomes related to ChatGPT (top two box scores and average values).

Using ChatGPT may have several benefits for students, as they may receive personalized instruction and feedback as well as support for various types of academic tasks [56]. However, there are also concerns about the limitations, challenges, and possible negative effects of ChatGPT use, such as student access to unreliable information and overreliance on the technology [56, 127, 128]. In our study, the average level of student agreement regarding the benefits of using ChatGPT for various learning tasks and outcomes ranged from 3.11 (facilitating completion of internship and improving employability) to 3.75 (improving general knowledge) (Fig 15). Most students tended to agree that ChatGPT could improve their general knowledge, with nearly 69%. This was followed by the view that ChatGPT could improve their specific knowledge and enhance their access to knowledge sources, both endorsed by nearly 63% of the students. Most students also agreed that ChatGPT could increase their study efficiency (59%), enhance their learning experience (58%), improve their ability to meet assignment deadlines, improve the quality of their assignments, and facilitate completing their studies, as emphasized by 57% of students.

**Fig 16:**
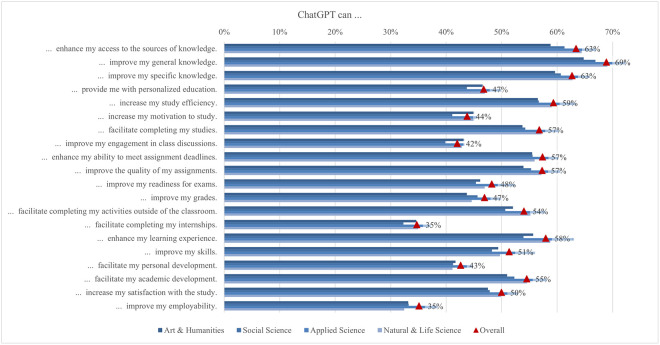
Differences in agreement on study issues and outcomes related to ChatGPT (top two box scores by field of study).

Students in all fields of study agreed at rates exceeding 50% that using ChatGPT could improve their general and specific knowledge, enhance their access to knowledge sources, their ability to meet assignment deadlines, and their learning experience, increase their study efficiency, improve their assignment quality, and facilitate completion of activities outside the classroom and their academic development (Fig 16). On the other hand, less than 40% of students in all disciplines thought that ChatGPT could help them complete their internships or improve their employability. Students in applied sciences exhibited the highest scores among disciplines for all statements on study-related issues and outcomes, while those in social sciences and in arts and humanities tended to show the lowest scores. The largest mean difference in agreement levels was currently observed in ChatGPT enhancement of learning experience, with a score of 0.20 higher for applied sciences compared to social sciences students. Other large and significant differences between the same group of students include completion of activities (0.19) and an increase in study efficiency (0.17). In addition, applied sciences students expressed more positive views than arts and humanities students about ChatGPT enhancing knowledge access (0.20), improving exam readiness (0.19), and improving grades (0.18). Finally, a large difference was found in improving employability, with a score of 0.19 higher for students in applied compared to those in natural and life sciences.

**Fig 17:**
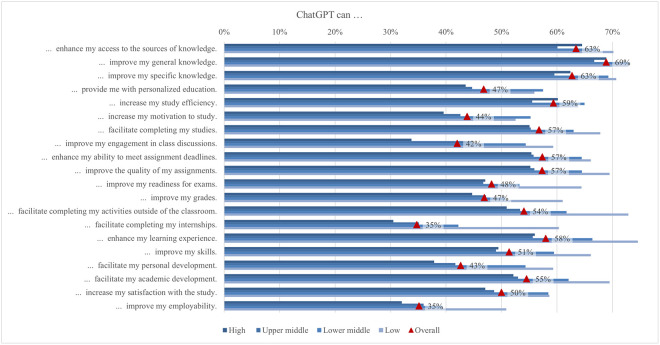
Differences in agreement on study issues and outcomes related to ChatGPT (top two box scores by income region).

Statistically significant differences in scores could also be observed when considering the income regions (Fig 17). Overall, students from low income regions tended to express higher levels of agreement with the statements compared to students from high income regions. The highest agreement scores for statements related to improving engagement in class discussions, facilitating internship completion, and supporting personal development were observed among students from low income regions, while the lowest scores were found among students from high income regions. The most significant differences between these groups were in the areas of improving class discussion engagement (0.62), facilitating internship completion (0.58), and supporting personal development (0.57). Independent of the income group, approximately 69% of students used ChatGPT to improve their general knowledge. Rural students exhibited significantly higher scores regarding ChatGPT facilitating their personal development and providing personal education compared to urban students, but the difference is small.

There were significant gender differences favoring male students in all statements. Regarding study issues, the largest mean score difference was found in providing students with personalized education and improving study efficiency (0.18). When examining statements relevant to student personal and professional development, we found the largest differenceđin improving employability (0.21). Many of the answers given to study issues and outcomes (for 11 out of 20 statements) were significantly affected by the level of study of the participating students. However, differences in mean scores tend to be small. In significant differences, outcomes of using ChatGPT were pointed to the greatest extent by first-level students. Almost all the possible study outcomes listed were selected in the smallest degree by third-level students. The difference determined by the level of study was the largest, 0.28 (the mean score is 3.37 for first-level students and 3.09 for third-level students), regarding the possibility of ChatGPT improving student’s grades. For all other potential study outcomes, the difference varied between 0.01 (facilitating personal development) and 0.19 (improving the quality of assignments).

#### Skills development

**Fig 18:**
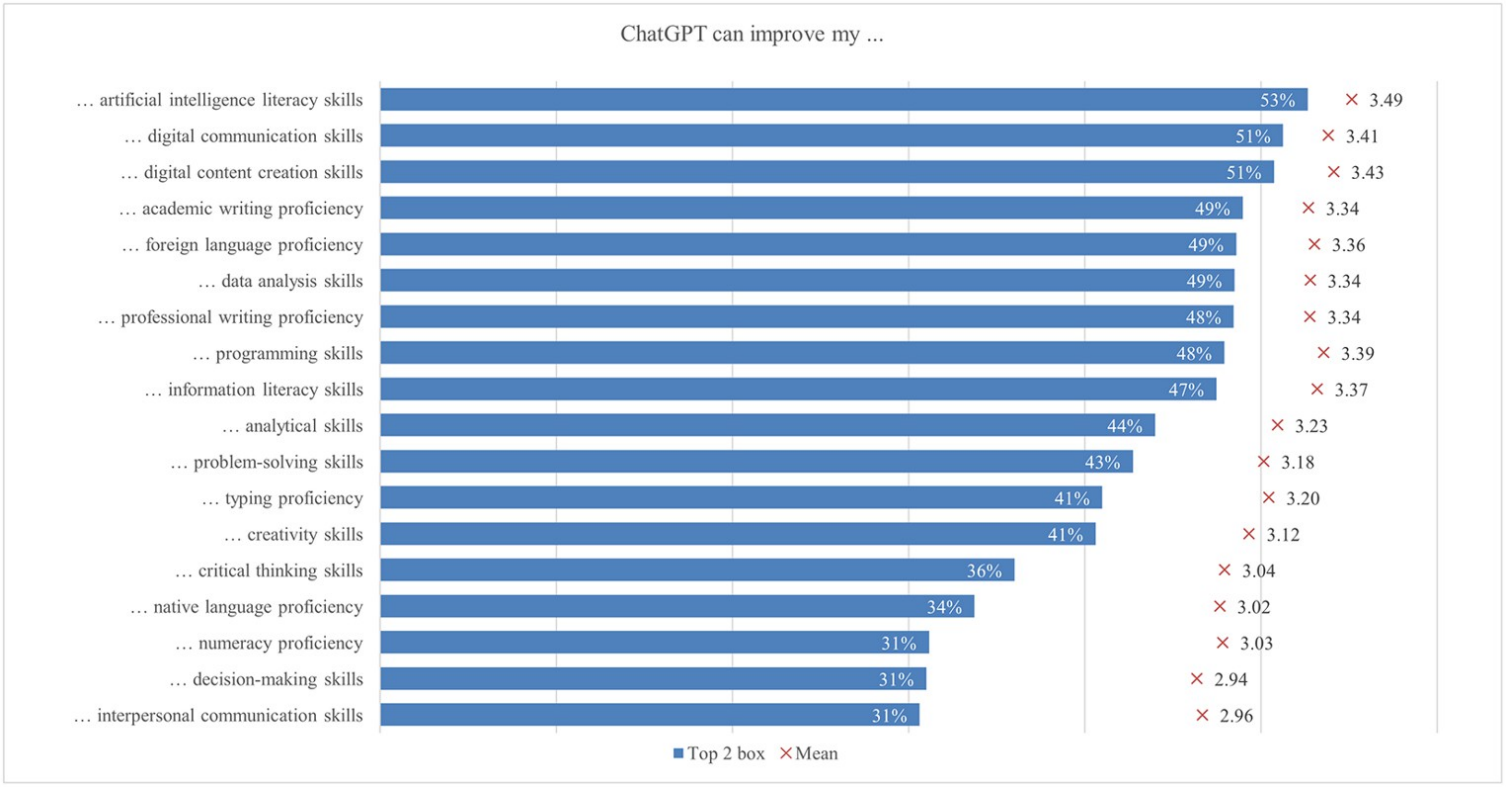
Agreement on the ChatGPT potential for skills development (top two box scores and average values).

Skills development using a single powerful instrument, such as ChatGPT, could provide a revolutionary alternative to traditional learning [6, 129–131]. Bitzenbauer [132] discusses some fascinating applications of such inventive learning in the development of skills required to comprehend complicated subjects in the classroom. Our results revealed ChatGPT’s potential effectiveness in enhancing various skills, though to varying extents, as also suggested by the previous research [133–136]. More than 50% of students agreed that ChatGPT has the potential to improve their AI literacy, digital communication, and digital content creation skills (Fig 18). Conversely, less than 40% of students agreed that ChatGPT has the potential to improve interpersonal communication, decision-making skills, numeracy, native language proficiency, and critical thinking skills. The varying effectiveness of ChatGPT in enhancing different skills is due to its strong alignment with digital skills, which involve information processing and content generation. In contrast, interpersonal communication, decision-making, and critical thinking require nuanced human interactions, emotional intelligence, and higher-order cognitive processes that are challenging for AI to fully replicate. Additionally, learning these complex skills often benefits from diverse, hands-on experiences and human feedback, which ChatGPT may not adequately provide.

**Fig 19:**
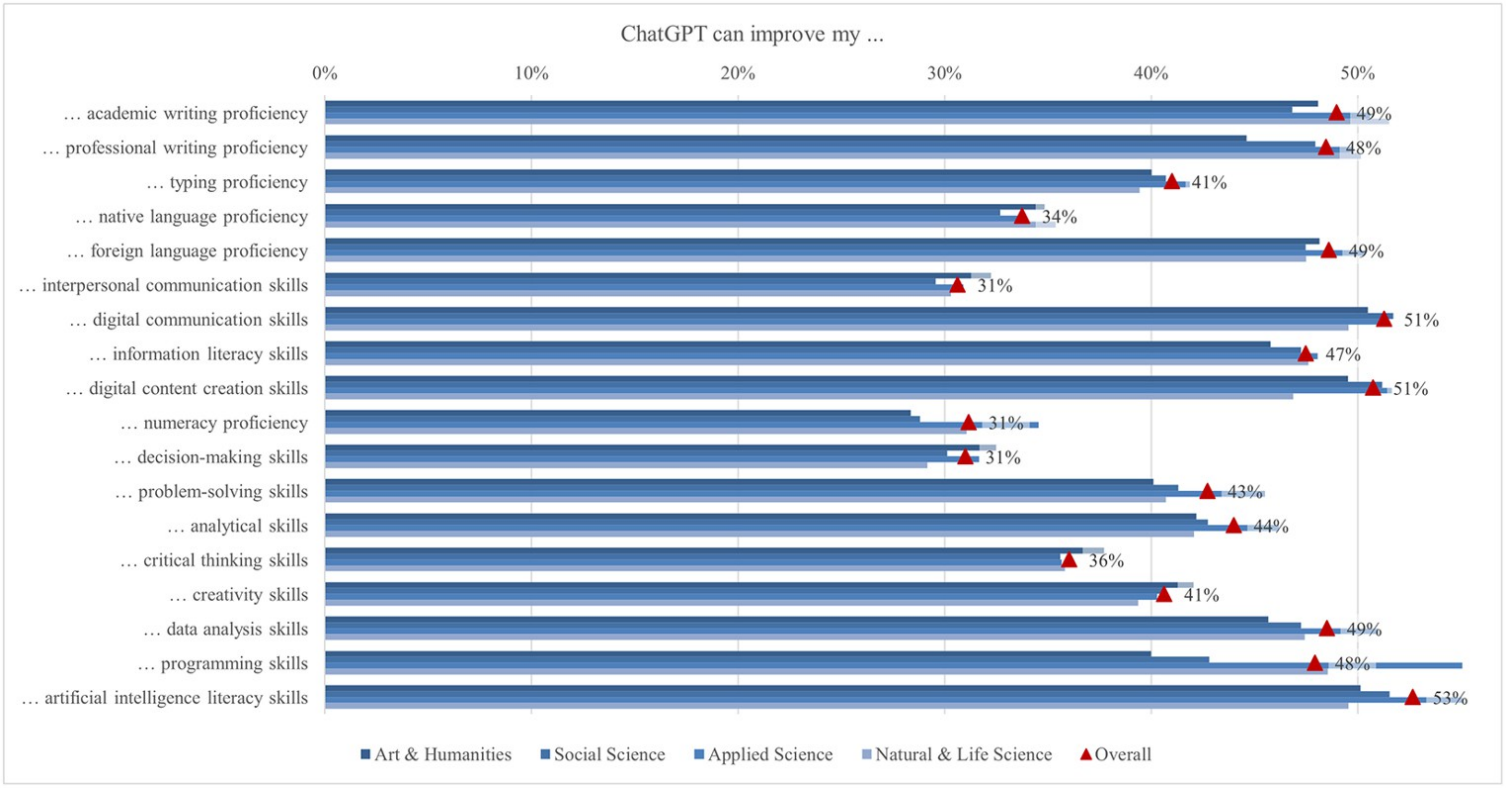
Differences in agreement on the ChatGPT potential for skills development (top two box scores by field of study).

The perceptions of ChatGPT’s potential to facilitate skills development varied significantly by field of study, impacting all skills except creativity (Fig 19). Overall, applied sciences students viewed ChatGPT as having a higher potential for facilitating the development of various skills compared to students from other fields. This was especially true for programming skills, where the difference between applied sciences students and arts and humanities students was most pronounced (0.34), followed by AI skills (0.16) and numeracy proficiency (0.14). However, despite generally perceiving a lower overall potential for skills development, arts and humanities students believed ChatGPT could significantly enhance interpersonal, critical thinking, and decision-making skills. The variations in perceptions of ChatGPT’s potential to facilitate skills development across different fields are influenced by the nature of the skills emphasized in each field. Applied sciences students viewed ChatGPT as highly beneficial for technical skills like programming, AI, and numeracy due to its ability to provide concrete assistance. In contrast, arts and humanities students, despite perceiving a lower overall potential for skills development, recognized ChatGPT’s significant enhancement of interpersonal, critical thinking, and decision-making skills, which are crucial in their curricula.

**Fig 20:**
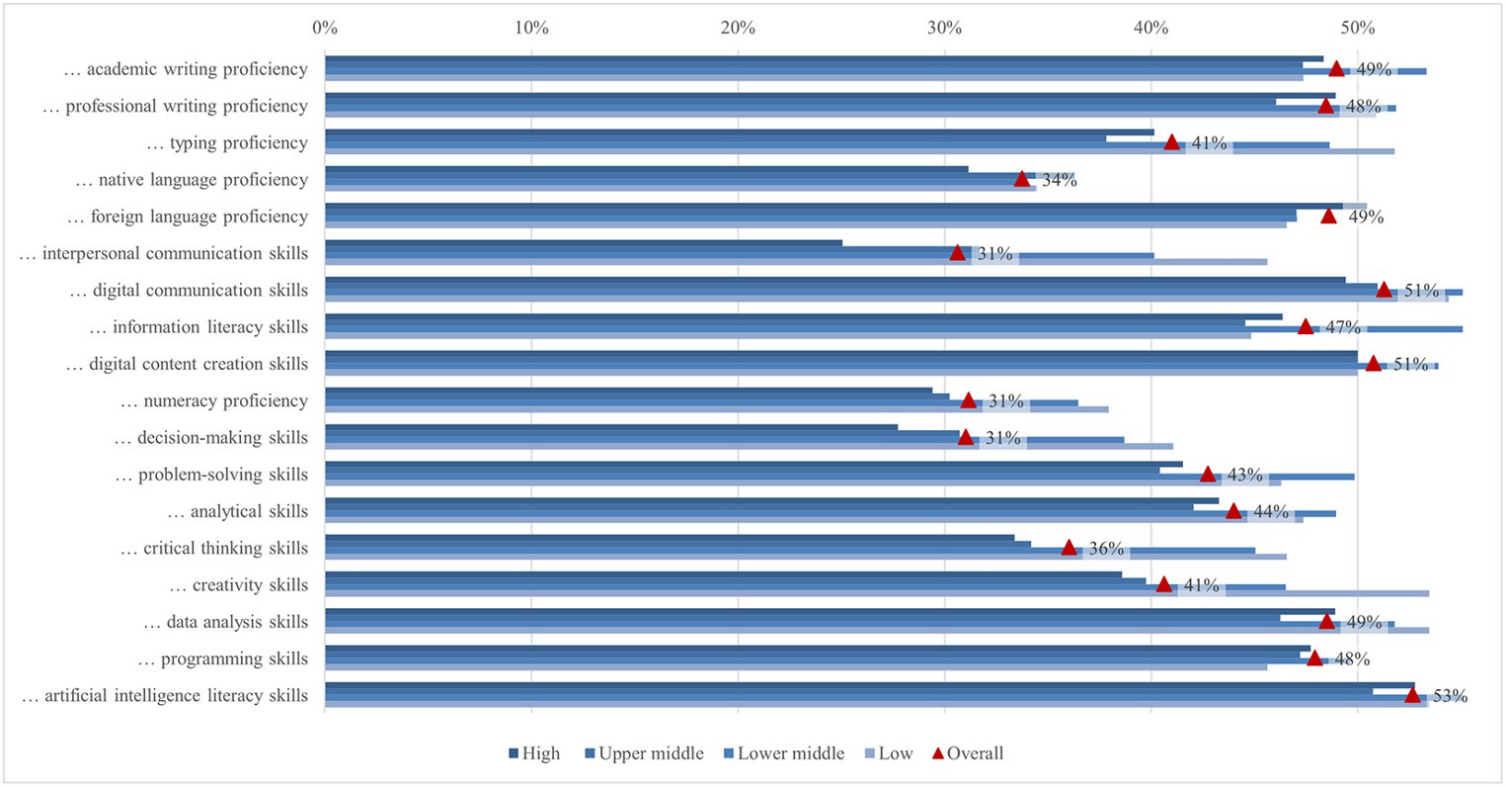
Differences in agreement on the ChatGPT potential for skills development (top two box scores by income region).

In general, students from lower and lower middle income regions found more potential in ChatGPT for skills development compared to students from high and upper middle income regions, particularly regarding interpersonal communication (0.62), critical thinking (0.57), decision-making (0.47), and creativity skills (0.46), except for facilitating foreign language proficiency, which was not significant (Fig 20). The variance in perception of ChatGPT’s potential for skills development between students from different income regions could be attributed to disparities in access to educational resources and support systems. Students from high and upper middle income regions often have access to private tutoring, advanced technology, and well-funded schools, reducing their reliance on tools like ChatGPT. Conversely, students from lower and lower middle income regions may find ChatGPT particularly valuable for developing skills such as interpersonal communication, critical thinking, decision-making, and creativity, as they have fewer alternative resources.

The rest of the demographic factors present insightful results. Contrasting to findings from Hu et al. [137], Park [49], Strzelecki [36], and Xu et al. [53], in our research, statistically significant differences according to gender have been observed. Males perceived ChatGPT to provide more overall support than females in various types of skills development, except for academic writing, typing proficiency, and digital communication abilities, where the results were not significant. Resonating with Moro-Egido’s [138] findings, gender differences were more pronounced in analytical and problem-solving skills, while communication skills showed comparable values across genders. The level of study revealed differences among students, aligning with Hu et al. [137] and Xu et al. [53]. First-level students tended to state that ChatGPT provides stronger support for analytical and problem-solving skills, whereas third-level students saw its potential in developing academic writing and foreign language proficiency. Regarding the mode of study, online and blended learning students generally perceive greater potential in ChatGPT for skills development compared to those in traditional modes, aligning with Jiang and Cheong [139]. Moreover, students in rural areas saw greater potential in ChatGPT for skills development, except for academic writing proficiency, which was more emphasized by urban students. In terms of economic position, an interesting “u-curve” is observed, with ‘significantly below average’ and ‘significantly above average’ having the greatest values and ‘average’ frequently in the middle. This is evident in interpersonal communication, native language proficiency, decision-making, and critical thinking skills. This perspective adds to studies such as Scherr et al. [140], which identified ChatGPT as increasingly useful for lower economic statuses.

#### Labor market and skills mismatch

**Fig 21:**
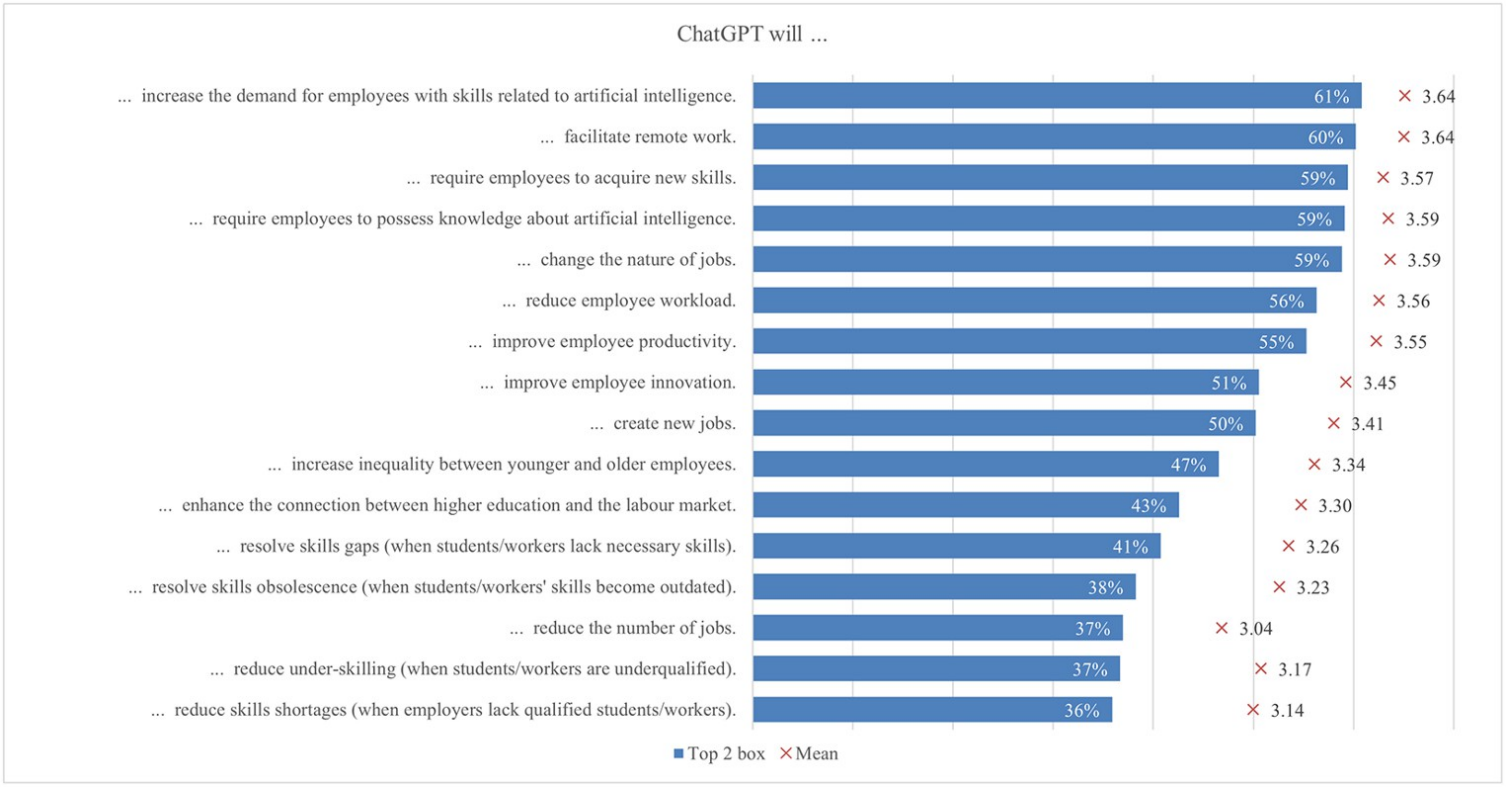
Agreement on the ChatGPT potential for labor market and skills mismatch (top two box scores and average values).

AI, including ChatGPT, as an important component of technological change, has various impacts on the labor market [83, 87, 141]. In our survey, students were asked about general challenges in the labor market connected with ChatGPT and the ability of ChatGPT to address potential skills mismatch (Fig 21). Students believed that the wide implementation of AI is likely to modify the future labor market. The highest percentage of students agreed that ChatGPT increases the demand for employees with AI-related skills (61%), facilitates remote work (60%), and requires employees to acquire new skills (59%). Besides, students believed that ChatGPT changes the nature of jobs (59%), requires employees to possess knowledge about AI (59%), which is consistent with the findings of Acemoglu et al. [141], Zarifhonarvar [142] and Cedefop [143]. However, students did not consider that implementing AI in various economic sectors could affect the unemployment rate in the labor market (37%). Consequently, the findings are in line with other studies suggesting that the primary impact of AI is to redesign parts of the working tasks rather than replacing entire jobs [144]. Due to changing tasks and the emergence of new job roles, workers need to acquire new or updated skills [143, 145, 146]. Regarding other aspects of the labor markets, only a minority of students considered that ChatGPT enhances the connection between higher education and the labor market (43%) and increases inequality between younger and older employees (47%), contradicting previous findings that jobs of young people are most exposed to automation [145]. Regarding skills mismatch, only a minority of students agreed with the statement that ChatGPT resolves skills gaps (41%) and skills obsolescence (38%), and reduces under-skilling (37%), and skill shortages (36%). However, the research by Komp-Leukkunen [87] has shown an ambivalent scenario where ChatGPT can replace software engineers to a large extent.

**Fig 22:**
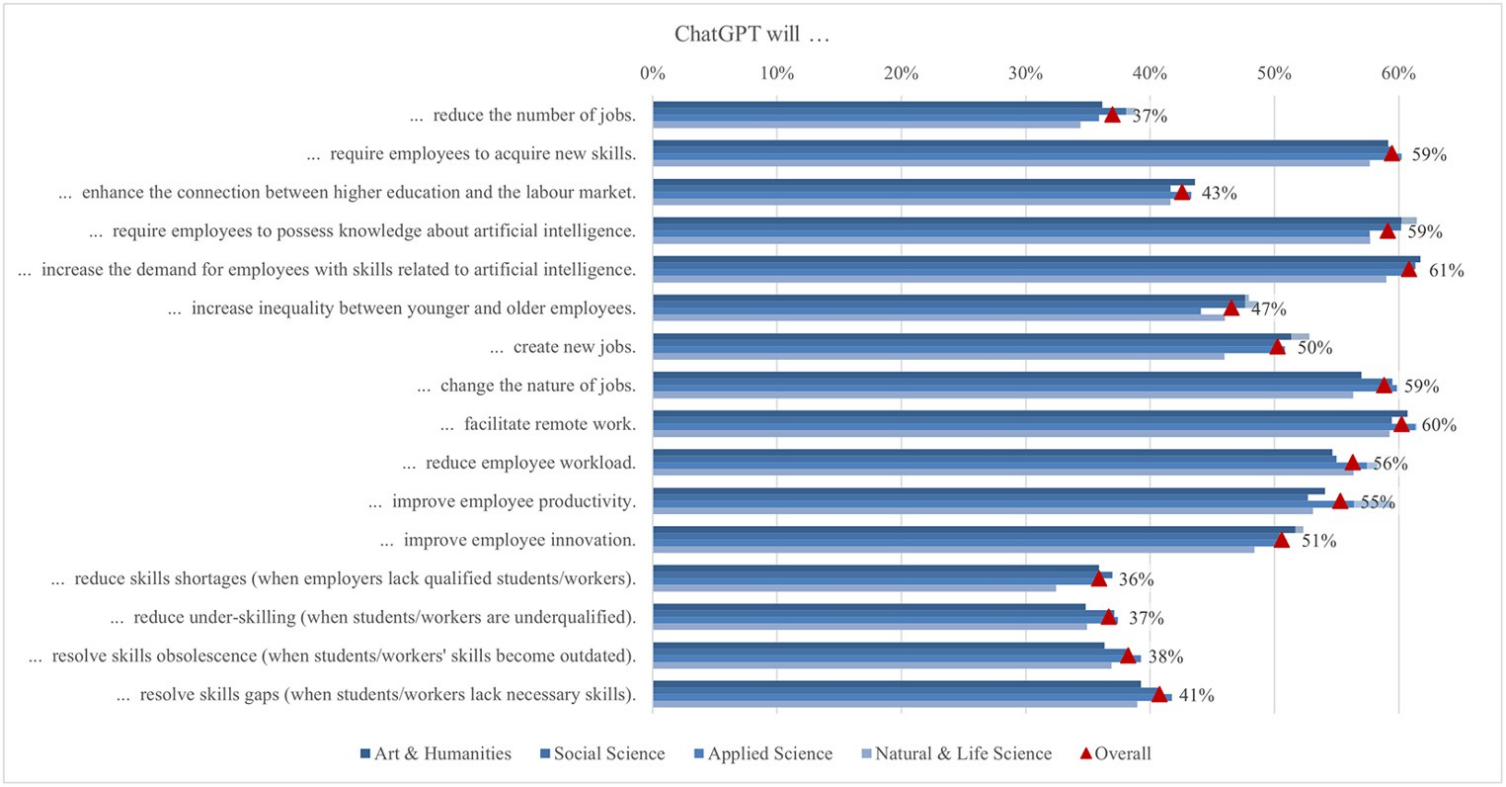
Differences in agreement on the ChatGPT potential for labor market and skills mismatch (top two box scores by field of study).

Ratings of challenges in the labor market of ChatGPT between representatives of different fields of study varied from 2.97 to 3.63 (Fig 22). The largest difference between fields of study concerned the statement that ChatGPT improves employee productivity (0.13) between students of applied sciences and social sciences. Arts and humanities students frequently noted that ChatGPT increases the demand for employees with AI-related skills (62%) and requires AI knowledge (62%). The popularity of AI knowledge requirements varied between 58% and 62%, depending on the field. Applied sciences students viewed ChatGPT most positively, citing benefits like facilitating remote work (61%), requiring new skills (60%), changing job nature (60%), improving productivity (59%), and reducing workload (58%), but they were less positive about reducing inequality (44%), resolving skills gaps (42%), and addressing skills shortages (37%). Social sciences students were positive about increased demand for AI-related skills (61%) and reduced skills shortages (37%) but concerned about increased inequality (49%) and job reduction (39%). They saw improved productivity (53%) as a moderate benefit. Arts and humanities students were optimistic about ChatGPT creating new jobs (53%), improving innovation (52%), and connecting education to the labor market (44%), but skeptical about reducing the workload (55%), resolving skill obsolescence (36%), and reducing underskilling (35%). Natural and life sciences students were generally negative, with fewer seeing benefits like job creation (46%) and innovation improvement (48%). The smallest perception gap was between applied sciences and natural and life sciences students (0.06), with over 40% using ChatGPT across fields. Differences likely stem from varying views on ChatGPT’s role in developing critical thinking skills [76].

**Fig 23:**
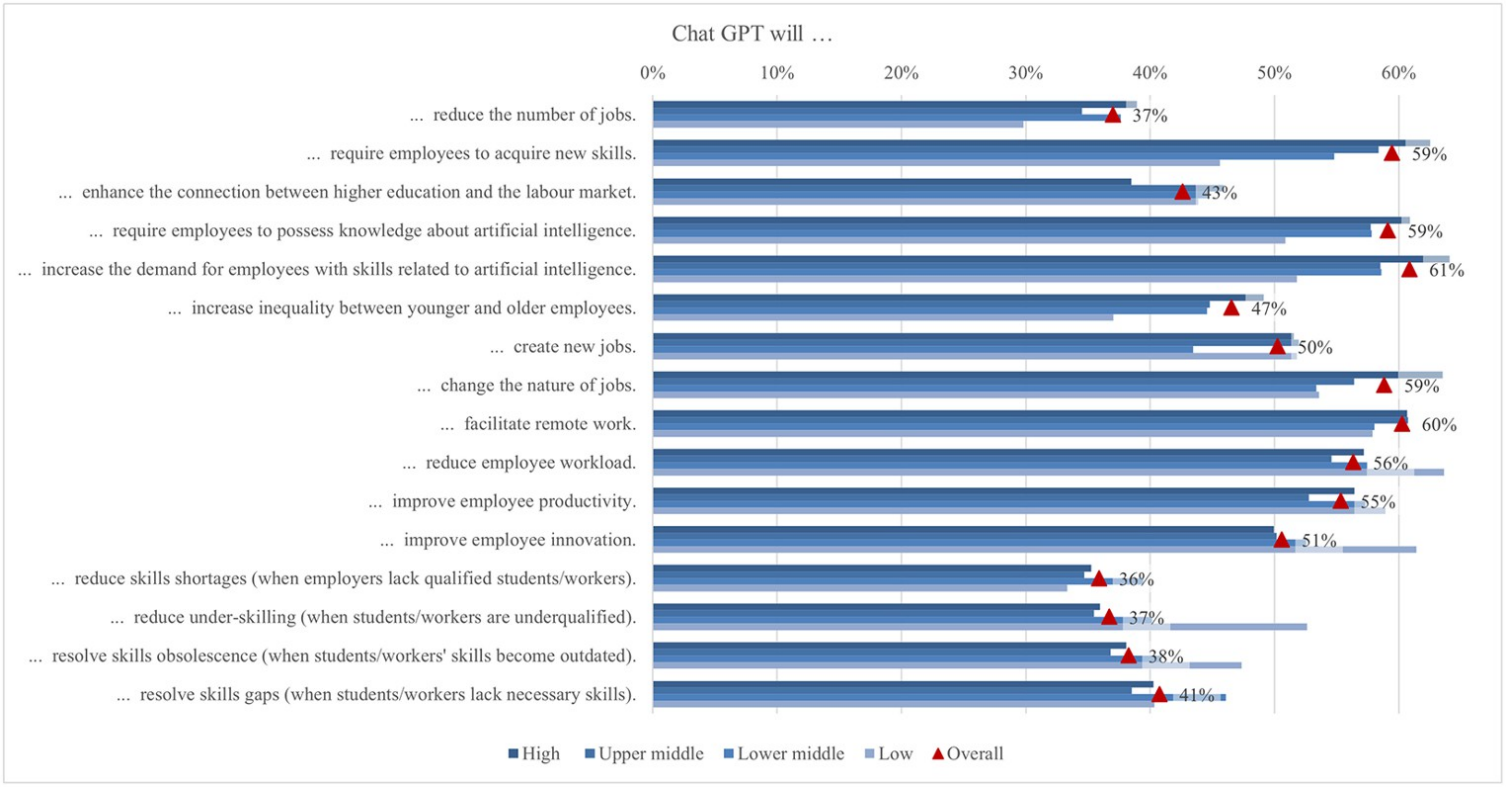
Differences in agreement on the ChatGPT potential for labor market and skills mismatch (top two box scores by income region).

Concerning income region, students most often indicated that ChatGPT increases demand for employees with skills related to AI, namely the highest from the high income regions (64%) and the lowest from the low income (52%) (Fig 23). Students from high income regions believed that ChatGPT would require employees to acquire new skills, while students in the upper middle income regions were facilitating remote work (61%), but students from the low income regions most frequently declared that ChatGPT reduces employee workload (64%). The two largest differences between high and low income regions were found in the agreement that ChatGPT has an impact on: (1) the need for new skills (0.30), where 63% of high income students agreed, and only 46% of low income students agreed, and (2) improving employee innovation (0.23), where low income students (61%) had higher confidence than high income students (50%).

Gender differences showed that males were more likely than females to select ‘agree’ or ‘strongly agree’ for all statements with statistically significant differences. This may reflect greater awareness among male students of the impact on the future labor market. Significant differences in terms of the level of study could be seen in only half of the statements. Specifically, the most significant difference was observed in the challenge that ChatGPT alters the nature of jobs (0.24) and necessitates employees to acquire new skills (0.27), with third level students experiencing this impact more strongly compared to those at the first level. Regarding the area in which they live, particularly in relation to the labor market, the urban area consistently received the highest scores across all statements, while the rural area scored highest on skills mismatch, but only in five statements.

#### Emotions

**Fig 24:**
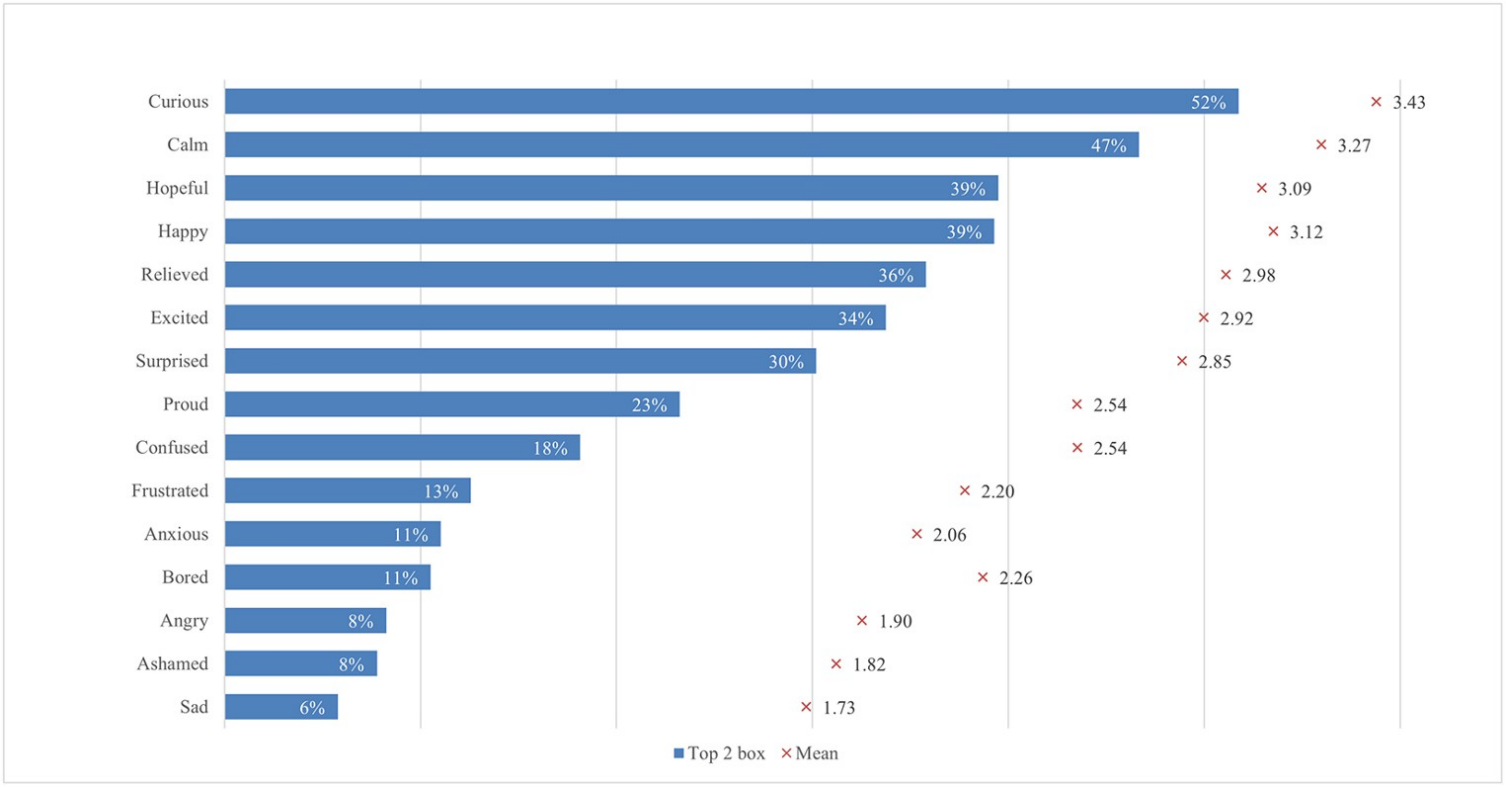
Frequency of emotions felt when using ChatGPT (top two box scores and average values).

ChatGPT has been depicted by previous studies as a tool that arouses mixed feelings in its users [22, 92, 93]. Therefore, our research aimed to explore also how often students felt eight positive (i.e., hope, calmness, relief, happiness, pride, surprise, curiosity, excitement) and seven negative emotions (i.e., boredom, sadness, shame, anger, anxiety, confusion, frustration) while using ChatGPT (Fig 24). On average, positive emotions ranged from 2.54 to 3.43, and negative emotions from 1.73 to 2.54. It can be concluded that the students do not experience a big change in their emotions. In most cases, they answered rarely or sometimes on average. The most frequently experienced emotions were curiosity (52%), calmness (47%), hope (39%), and happiness (39%), while sadness (6%), shame (8%), and anger (8%) were those experienced least frequently. Among positive emotions, pride was the least frequent (23%), and among negative emotions, confusion was the most frequent (18%). Such results suggest that, overall, while using ChatGPT, students tend to feel more positively than negatively, which is also in line with previous findings [93]. Curiosity is the prevalent emotion, a result that can be explained by the relative novelty of this tool and AI in general.

**Fig 25:**
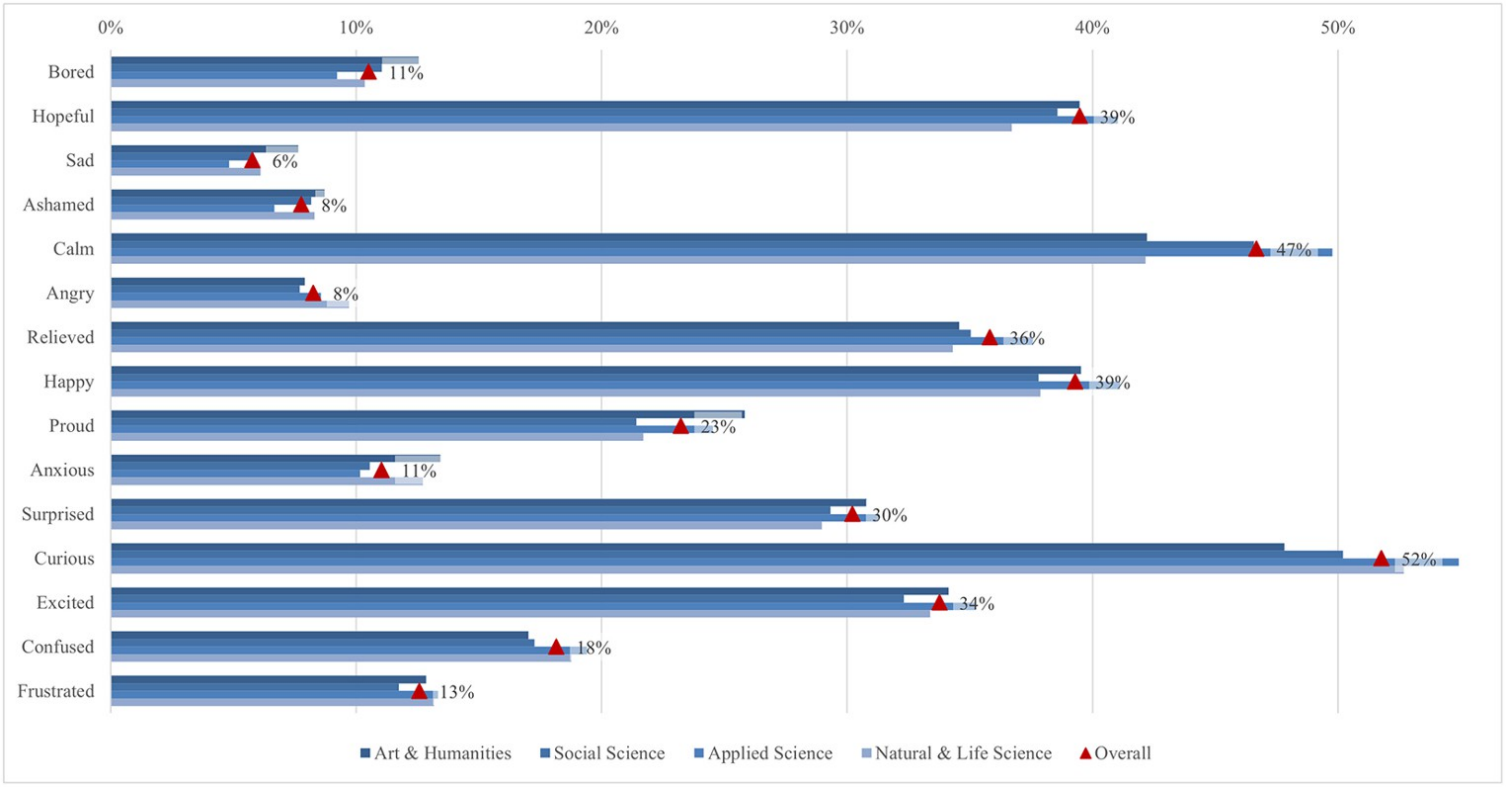
Differences in frequency of emotions felt when using ChatGPT (top two box scores by field of study).

The results comparing the emotions experienced by students from different fields of study when using ChatGPT show clear differences in percentage between positive and negative emotions, with a prevalence of the former, in general, tending to feel more positively than negatively (Fig 25). For all emotions considered, the percentages in the different fields of study were relatively uniform. Considering positive emotions, the highest percentages were found for students from applied sciences. For example, they got the highest percentages in feelings such as curiosity (55%), calmness (50%), happiness (41%), hope (41%), relief (38%), excitement (35%), and surprise (31%). Contrastingly, when considering negative feelings, data showed that students from arts and humanities seemed to be more likely to experience emotions such as anxiety (13%), boredom (13%), shame (9%), anger (8%), and sadness (8%) when using ChatGPT. Finally, it is important to mention that the largest difference between the fields of study was for calmness (0.21), with the highest values for applied sciences and the lowest for natural and life sciences. We could speculate that specific technical competences of students of the applied sciences could increase their perception of control when using ChatGPT and, in turn, favor their calmness. This interpretation should be further investigated by examining other data.

**Fig 26:**
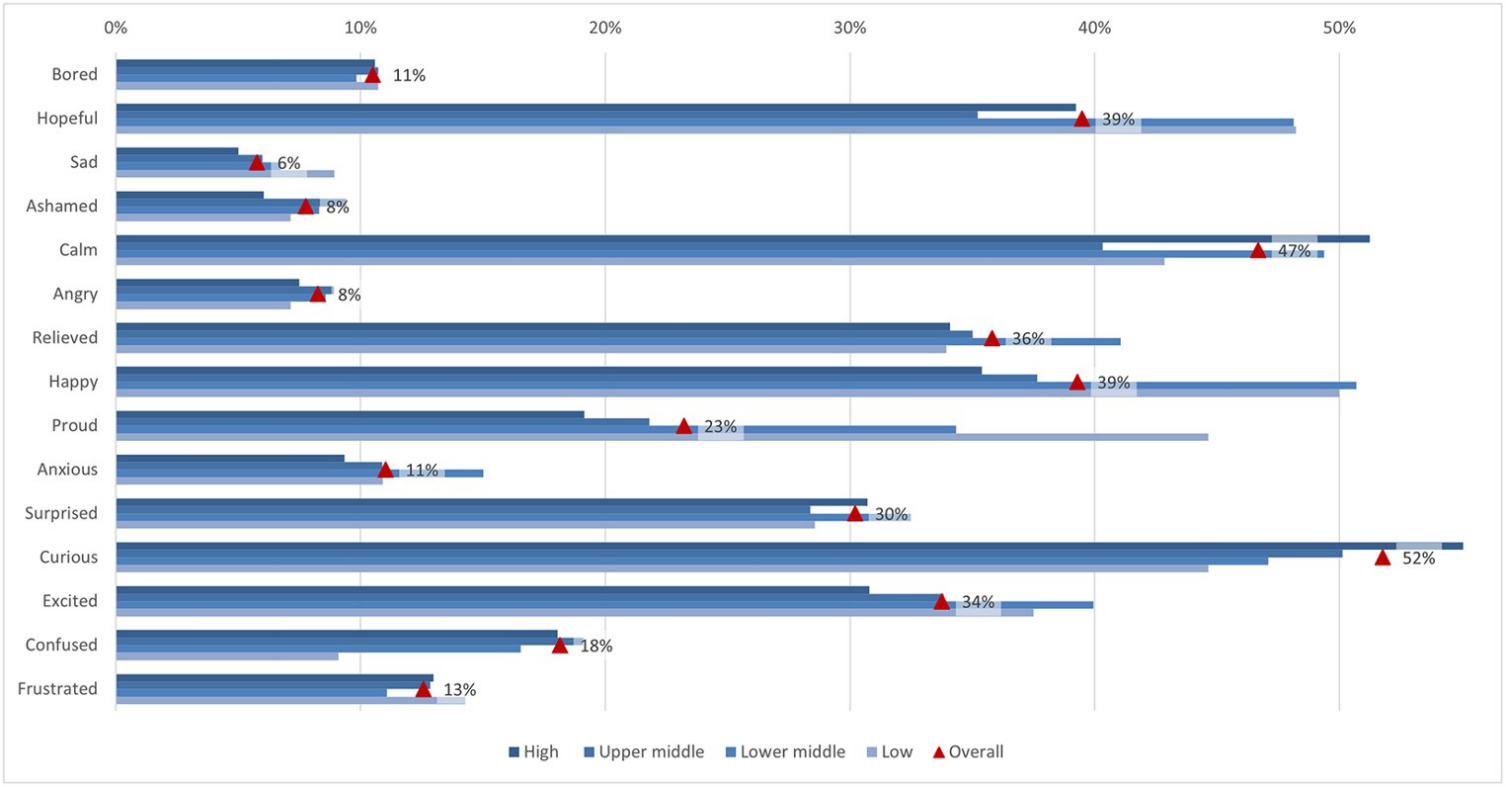
Differences in frequency of emotions felt when using ChatGPT (top two box scores by income region).

In addition, the results showed notable differences between the specific type of positive emotions experienced depending on the income region type (Fig 26). Interestingly, low and lower middle income regions, compared with high and upper middle income regions, experienced more frequently activating positive emotions such as happiness and excitement. Also, students from low and lower middle income regions, compared with those from high and upper middle income regions, reported to feel more hopeful and proud. On the other hand, the most frequently experienced emotions for high and upper middle income regions were curiosity (55% and 50%) and calmness (51% and 40%). Overall, these results indicate that the experience associated with the use of ChatGPT is generally positive, generating primarily positive rather than negative emotions in students, regardless of income. However, using AI might be more stimulating for lower than higher income groups as they probably are less likely to be exposed to these types of experiences.

The results comparing the emotions experienced by students from different modes of study showed that students from traditional, online, and blended modes felt mostly positive emotions. However, we found out that students involved in traditional learning contexts usually felt worse than others when using ChatGPT despite experiencing anxiety less frequently. Living in urban areas affected students’ emotions in mixed ways. Negative emotions such as boredom, sadness, shame, anger, anxiety, confusion, and frustration were significantly lower for students living in urban areas compared to those living in rural or suburban areas; nevertheless, the same trend was also found for positive emotions such as hope, calmness, happiness, pride, and surprise, with lower scores for students living in urban areas. These findings could suggest a somewhat more intense emotional engagement for these students, combining together both positive and negative feelings with related effects. However, students who lived in urban areas, as well as suburban and rural areas, declared that they experienced positive feelings more frequently than negative feelings; for all of them, the most frequent emotions were calmness, happiness, and hope. Finally, regarding gender, females tended to show higher levels of positive emotions and lower levels of negative emotions compared to males, with some exceptions (i.e., anger and frustration).

### Regression analysis results

Ordinal logistic regression was utilized to empirically validate the impact of various factors associated with ChatGPT aspects on students’ usage patterns for specific academic tasks, particularly those most frequently used by students, such as brainstorming, summarizing, and academic writing, as suggested by the highest mean values from the first part of the analysis. Therefore, different ordinal regression models (Eq 1) with three dependent variables were estimated (*Y*_1_ = *Brainstorming*, *Y*_2_ = *Summarizing* and *Y*_3_ = *Academic writing*) with 14 main independent variables of interest (*X*_1_ = *Simplify complex information*, *X*_2_ = *Summarize extensive information*, …, *X*_14_ = *Calm*). Moreover, socio-demographic and geographic characteristics were used as control variables in all three estimated models. The models estimated the conditional probability P(Y_i_ ≤ j|X_1_, X_2_,…, X_p_) that dependent variables were less or equal to *j* given the values of independent variables (X_1_, X_2_, …, X_p_). The value *j* ranges from 1 to *k* − 1 where *k* is the number of ordered categories of the dependent variables *Y*_*i*_. In this case, there are 5 categories in the individual dependent variable (*k* = 5) as their values range from 1 = *never* 1 to 5 = *always*. Accordingly, the models estimate coefficients associated with independent variables (β_1*i*_, β_2*i*_, …, *β*_*pi*_) and the intercepts (*α*_*ji*_) for *j* = 1, 2, …, *k* − 1. Since the interpretation of control variables and intercepts is not the main focus in this context, they are omitted from the presentation of the ordinal regression results.


$$P\left(Y_{i}\leq j|X_{1},X_{2},\ldots ,X_{p}\right)=\frac{exp(\alpha_{ji}+\beta_{1i}X_{1}+\beta_{2i}X_{2}+\ldots +\beta_{pi}X_{p})}{1+exp(\alpha_{ji}+\beta_{1i}X_{1}+\beta_{2i}X_{2}+\ldots +\beta_{pi}X_{p})}for\;i=1,2,3$$ (1)

Prior to parameter estimation, two key assumptions of ordinal logistic regression were verified, namely the proportional odds assumption and multicollinearity. The proportional odds assumption was tested using the test of parallel lines, which was significant (p < 0.001) for all three estimated ordinal logistic regression models, indicating that the regression slopes differ significantly across the levels of the dependent variable for all models [147]. However, this test is considered anti-conservative because it almost always results in rejecting the proportional odds assumption [148], especially when there are many independent variables [149] or when the sample size is large [150]. Moreover, multicollinearity was tested using several approaches. Initially, a Spearman correlation between the independent variables presented in the supporting information (S1 Table) did not indicate any strong relationship between them, suggesting there were no issues of multicollinearity. The severity of multicollinearity was further tested using multicollinearity diagnostics. The VIF values ranged between 1.1 and 2.0, and TOL values ranged between 0.5 and 0.9. Since VIF should not exceed 10 and TOL should be above 0.1, all of the values were considerably within the acceptable thresholds, confirming the absence of multicollinearity [151].

Due to the complete case analysis approach adopted in the ordinal regression, the number of valid full student responses varied across the ordinal logistic regression models. The model predicting ChatGPT usage for brainstorming included 11,333 responses, for summarizing included 11,339 responses, and for academic writing included 11,343 responses. Assuming that the data were missing at random, parameter estimation was carried out. Finally, the goodness-of-fit statistics for the proposed empirical model proved to be adequate, as suggested by Nagelkerke R² values of 0.134, 0.170, and 0.156 for each model, respectively [152]. The results of the ordinal logistic regression are presented in Table 2.

**Table 2:** Ordinal logistic regression for factors influencing the ChatGPT usage.

ChatGPT aspect	Selected ChatGPT-related factor	ChatGPT Usage					
		Brainstorming		Summarizing		Academic writing	
		Coeff.	Std. Error	Coeff.	Std. Error	Coeff.	Std. Error
Capabilities	ChatGPT can simplify complex information.	0.190***	0.027	0.220***	0.027	0.180***	0.027
	ChatGPT can summarize extensive information.	0.032	0.027	0.402***	0.027	0.160***	0.027
Regulation and ethical concerns	Students should take appropriate measures to protect their own personal information.	-0.104***	0.020	-0.132***	0.020	-0.222***	0.020
	ChatGPT should be subject to university/faculty ethical guidelines.	-0.002	0.017	-0.069***	0.017	-0.090***	0.017
Satisfaction and attitude	Using ChatGPT is interesting to me.	0.215***	0.025	0.150***	0.025	0.215***	0.025
	ChatGPT can help with things in everyday life.	0.163***	0.020	0.173***	0.020	0.123***	0.020
Study issues and outcomes	ChatGPT can enhance my access to the sources of knowledge.	-0.019	0.026	-0.057*	0.026	0.108***	0.026
	ChatGPT can improve my general knowledge.	0.124***	0.028	0.085**	0.028	0.105***	0.028
Skills development	ChatGPT can improve my artificial intelligence literacy skills	0.079***	0.021	0.104***	0.021	0.136***	0.022
	ChatGPT can improve my digital content creation skills	0.130***	0.022	0.103***	0.022	0.089***	0.022
Labor market and skills mismatch	ChatGPT will increase the demand for employees with skills related to artificial intelligence.	0.048*	0.022	0.004	0.022	0.025	0.022
	ChatGPT will facilitate remote work.	0.027	0.022	0.008	0.022	-0.015	0.022
Emotions	Curiosity	0.107***	0.017	0.075***	0.017	0.008	0.017
	Calmness	0.069***	0.015	0.067***	0.015	0.072***	0.015
	Nagelkerke R^2^	0.134		0.170		0.156	
	N	11,333		11,339		11,343	

Note:

* p < 0.05;

** p < 0.01;

*** p < 0.001.

The results of the ordinal logistic regression revealed key insights into the influence of selected factors related to ChatGPT on students’ usage patterns across different use cases, such as brainstorming, summarizing, and academic writing. Specifically, the ability to simplify complex information consistently showed significant positive effects on the frequency of using ChatGPT across all three use cases, while the ability to summarize extensive information had a significant positive impact on two of the use cases. Both factors had the most substantial impact on the frequency of using ChatGPT for summarizing (β = 0.220; p < 0.001 and β = 0.402; p < 0.001, respectively). Conversely, the potential of ChatGPT to enhance access to sources of knowledge appeared to have a negative and statistically significant impact on using ChatGPT for summarizing (β = -0.057; p < 0.05), while it had a positive and statistically significant impact on academic writing (β = 0.108; p < 0.001). Additionally, the potential of ChatGPT to improve general knowledge had a positive and significant effect on the frequency of using ChatGPT across all three use cases, with the highest impact observed in brainstorming (β = 0.124; p < 0.001). This was likely because simplifying complex information and summarizing extensive information directly enhanced comprehension and efficiency, making these skills particularly valuable for summarizing tasks. In contrast, accessing knowledge sources might have introduced more information than necessary for summarizing, but it was beneficial for in-depth academic writing while improving general knowledge broadly supported brainstorming activities.

In most examined cases, regulation and ethical concerns seemed to have a negative impact on the frequency of ChatGPT use. Students’ belief that they should take appropriate measures to protect personal information had a negative and statistically significant impact on the frequency of using ChatGPT across all three use cases, with the strongest negative impact observed in academic writing (β = -0.222; p < 0.001). Despite still being statistically significant, the impact of the belief that ChatGPT should be subject to university/faculty ethical guidelines on academic writing and summarizing was not as prominent. Regulation and ethical concerns negatively impacted ChatGPT use among students, particularly in academic writing, due to heightened awareness and caution around data privacy and academic integrity. These concerns led students to limit their ChatGPT use, especially for tasks requiring originality and intellectual effort, to avoid potential academic misconduct and focus on their own development. Conversely, the impact on tasks like summarizing was less pronounced, indicating some comfort in using AI for less critical academic activities.

The perceived ability of ChatGPT to potentially facilitate skills development had significant implications for the frequency of its use in all three use cases. The most pronounced effects were observed in two specific instances. The first instance was the impact of ChatGPT’s perceived ability to enhance AI literacy skills, which was associated with an increased frequency of using ChatGPT for academic writing (β = 0.136; p < 0.001). The second instance was the impact of ChatGPT’s perceived ability to enhance digital content creation skills, which was associated with an increased frequency of using ChatGPT for brainstorming (β = 0.130; p < 0.001). On the other hand, the least prominent effects were observed for ChatGPT’s perceived ability to enhance AI skills on the use of ChatGPT for brainstorming (β = 0.079; p < 0.001) and its perceived ability to enhance digital content creation skills on academic writing (β = 0.089; p < 0.001). However, regarding labor market and skills mismatch, there was only one instance where the perceived potential of ChatGPT to increase demand for employees with AI-related skills had a significantly positive effect on using ChatGPT for brainstorming (β = 0.048; p < 0.05). The varying impacts of ChatGPT’s perceived ability to facilitate skills development depended on how well the skills aligned with specific activities. Users who saw ChatGPT as enhancing AI literacy used it more for academic writing, while those who believed it improved digital content creation used it more for brainstorming. Weaker effects were observed when the skills and activities were less directly related, while the labor market effect suggested users utilized ChatGPT for brainstorming to develop marketable AI skills.

Finally, emotional factors played a significant role in determining the frequency of ChatGPT usage, with an exception in the context of the impact of curiosity on academic writing. Students who felt curious and calm while interacting with ChatGPT tended to use it more frequently, with curiosity having the highest observed effect on the use of ChatGPT for brainstorming (β = 0.107; p < 0.001), while its impact on other use cases was less pronounced. The exploratory nature of brainstorming aligned well with curiosity, leading to this high impact, while it had a lesser effect on using ChatGPT for academic writing, which requires a more structured and focused approach. Additionally, feeling calm when using ChatGPT generally enhanced user engagement across various tasks.

## Discussion

The introduction of ChatGPT in November 2022 marked a pivotal moment for the integration of AI in higher education. Within its first year, ChatGPT gained widespread popularity among students due to its advanced natural language processing capabilities, which enable smooth and intuitive user interactions [153]. In order to capture early perceptions of higher education students, a global survey was conducted between October 2023 and February 2024, offering a comprehensive global perspective on its initial acceptance and potential impact within educational and broader contexts from a students’ point of view. After a year and a bit more of ChatGPT’s existence, most students have used ChatGPT, which points to its popularity within the higher education context.

Students used ChatGPT mainly for brainstorming, summarizing texts, finding research articles, and writing. These use cases align with the tool’s strengths in generating ideas, organizing content, and providing feedback on drafts. For instance, Menon and Shilpa [28] highlight that AI tools such as ChatGPT can assist in formulating research questions, summarizing information, and offering writing support in academic contexts. The less frequent use for professional and creative writing could be due to the personal and nuanced nature of these tasks, which often require a human touch. Students found ChatGPT particularly useful in simplifying complex information and summarizing extensive content, thereby enhancing their understanding and managing large volumes of information more efficiently and effectively. These findings are supported by Bouteraa et al. [23] and Chan and Hu [30], who noted the tool’s effectiveness in clarifying technical details and summarizing vast amounts of data. However, students expressed concerns about the reliability of the information provided by ChatGPT and its support for traditional classroom learning, echoing sentiments in studies by Biswas [31] and Mai et al. [108]. There is a unanimous agreement among students on the need for AI system regulations at various levels (international, national, organizational, and faculty) due to concerns about ChatGPT promoting cheating, plagiarism, and social isolation. These concerns are well-documented in the literature. For instance, AlAfnan et al. [1] and Fütterer et al. [22] discuss the ethical implications and potential misuse of ChatGPT in academic settings, emphasizing the importance of establishing clear guidelines and regulatory frameworks.

In everyday life, students generally found ChatGPT interesting and useful, with over half valuing its control and assistance features. However, some students preferred information from peers and teachers, which indicates a preference for human expertise and interaction over AI-generated content. This preference is discussed in studies by Sullivan et al. [9] and Castonguay et al. [154], which highlight the mixed feelings students have towards AI tools in educational settings. In our research, ChatGPT was perceived to enhance students’ knowledge, access to knowledge sources, learning experiences, study efficiency, and chances of getting good grades. Most students agreed that ChatGPT could improve their general and specific knowledge, enhance their learning experience, and increase their study efficiency. This is supported by findings from Strzelecki [36], which emphasizes the tool’s potential to positively impact academic performance when used appropriately.

Students viewed ChatGPT as an efficient tool for potentially improving AI literacy, digital communication, and digital content creation skills. However, its effectiveness was less prominent in enhancing interpersonal communication, decision-making skills, numeracy, native language proficiency, and critical thinking skills. Studies by Tiwari et al. [29] and Chiu [18] support these findings, highlighting ChatGPT’s strengths in digital competencies while noting the areas where traditional learning methods remain superior. Regarding the labor market, students from our research believed that AI would increase the demand for related skills and facilitate remote work without significantly affecting unemployment rates. They emphasized the importance of acquiring new skills to remain competitive in a technologically evolving job market. Research by Chen et al. [83] and Komp-Leukkunen [87] indicates that jobs involving writing and programming are more susceptible to being impacted by AI, underscoring the need for new competencies in the workforce. Emotionally, students generally felt more positive than negative while using ChatGPT, with curiosity and calmness being the most common emotions. This positive emotional response suggests that students are open to integrating AI tools like ChatGPT into their academic and daily routines. Abbas et al. [91] and Mamo et al. [93] report similar findings, noting that the immediate benefits and convenience of using ChatGPT contribute to its positive reception among students.

However, students’ perceptions of various ChatGPT aspects were found to differ across fields of study and income regions, as well as other selected socio-demographic and geographic characteristics, with the most notable differences further discussed. Applied sciences students showed higher satisfaction with ChatGPT, appreciating its capabilities in simplifying complex information and providing clear technical support. They found ChatGPT particularly useful for technical communication and problem-solving tasks. In contrast, students in the arts and humanities were more critical of ChatGPT’s ability to handle nuanced and interpretive language, reflecting a preference for human interaction in these fields. Social sciences students also exhibited lower satisfaction with ChatGPT compared to their peers in applied sciences due to the subjective analysis required in their disciplines [23]. Perceptions of ChatGPT also varied significantly across income regions. Students from low income regions tended to express higher agreement with the benefits of ChatGPT, particularly in improving class discussion engagement, facilitating internship completion, and aiding personal development. These students relied more on cost-effective digital tools like ChatGPT to supplement their limited educational resources. Conversely, students from high income regions valued ChatGPT’s innovative aspects and its capability to understand and follow human instructions, indicating a higher familiarity and comfort with advanced technological tools, as pointed out by Han and Kumwenda [109] or Alharbi [110].

Gender differences were evident in students’ perceptions of ChatGPT. Male students generally reported higher levels of satisfaction and more positive attitudes towards ChatGPT compared to female students. This difference might be attributed to a greater comfort and familiarity with technology among male students. Female students, on the other hand, expressed more concerns about the ethical implications and potential misuse of ChatGPT, such as facilitating plagiarism and spreading misinformation, which is in line with the findings of Xu et al. [53] and Schepman and Rodway [126]. The level of study also influenced students’ perceptions of ChatGPT. First level students were more likely to perceive ChatGPT as beneficial for their academic development, improving grades, and enhancing study efficiency. This can be attributed to their greater need for academic support and their openness to adopting new technologies. In contrast, higher level students who have developed their own learning strategies and have more experience with academic tools tend to be more critical of ChatGPT’s accuracy and reliability, aligning with the findings of Kelly et al. [155] and Xu et al. [53]. Students engaged in online and blended learning programs reported finding ChatGPT more useful and easier to interact with than those in traditional learning environments. This may be due to the higher integration of digital tools in online education, which aligns well with ChatGPT’s functionalities. As suggested by Jiang and Cheong [139], these students appreciate the personalized learning experience and real-time feedback provided by ChatGPT, which enhances their learning outcomes and study efficiency. Students from rural areas demonstrated higher usage of ChatGPT and perceived greater benefits from its use compared to urban students. This higher perception among rural students is likely due to limited access to educational resources, making ChatGPT a valuable tool for supplementing their learning. However, the differences between urban and rural students’ perceptions were generally small, similarly as noted by Javaid et al. [60]. Economic status significantly shapes students’ perceptions of ChatGPT. Students from economically disadvantaged backgrounds tended to use ChatGPT more frequently and reported higher levels of satisfaction with its capabilities, particularly in enhancing their study efficiency and providing personalized support. In contrast, students from higher economic backgrounds showed a greater appreciation for ChatGPT’s ability to handle complex instructions and its innovative features, reflecting their familiarity with advanced technological tools, as also pointed out by Scherr et al. [140].

Additionally, several other factors highlighted in the existing literature can explain students’ interaction and engagement with ChatGPT, including performance expectancy, effort expectancy, social influence, facilitating conditions, privacy concerns, perceived interactivity, perceived human touch, and more [28]. The results of the ordinal logistic regression in our research showed that various factors had significantly influenced ChatGPT usage across the tasks for which students used it most frequently, such as brainstorming, summarizing, and academic writing. Students who were more aware of ChatGPT’s capabilities, such as simplifying complex information and summarizing extensive content, more frequently used ChatGPT, particularly for summarizing. While ChatGPT’s potential to enhance access to knowledge negatively affected its use for summarizing, it positively impacted its use for academic writing, and its potential to improve general knowledge boosted usage across all tasks, especially brainstorming. Moreover, students who expressed greater regulation and ethical concerns about ChatGPT less frequently used ChatGPT across all tasks, particularly for academic writing, due to data privacy and academic integrity concerns. While the perceived ability of ChatGPT to facilitate skills development (AI literacy and digital content creation skills) positively affected the frequency of its use across all three cases, its perceived potential to increase demand for employees with AI-related skills had a positive effect only on using ChatGPT for brainstorming. Finally, emotional factors, such as curiosity and feeling calm, also enhance the frequency of students’ ChatGPT engagement, especially for brainstorming. The results confirm that students’ usage patterns of ChatGPT varied across different factors, supplementing the findings from other studies (e.g., Bouteraa et al. [23], Garrel & Mayer [107], Romero-Rodríguez et al. [156], Shoufan [85], Strzelecki [41]) that found relationships between usage and students’ personal traits, such as habit, hedonic motivation, and personal innovativeness.

## Conclusion

The global study reveals how higher education students perceive ChatGPT in its early stages. Students view ChatGPT as a valuable tool primarily for brainstorming, summarizing texts, and academic writing, appreciating its ability to simplify complex information. However, they express skepticism about its reliability and effectiveness in traditional classroom settings, raising concerns about cheating, plagiarism, and privacy issues. While students report a positive attitude towards ChatGPT, finding it interesting and helpful, they still prefer human interaction, underscoring the importance of personal connections in learning. They believe that ChatGPT can enhance study efficiency and knowledge acquisition but acknowledge the risks of dependency that may undermine critical thinking skills. Although it is seen as beneficial for developing AI literacy and digital content creation skills, it is less effective in fostering interpersonal communication. Students anticipate increased demand for tech-related skills in the labor market due to AI’s rise, highlighting the need for ongoing skill development without expecting significant impacts on unemployment rates. Overall, students experience positive emotions, such as curiosity and calmness, when using ChatGPT, indicating a readiness to integrate AI into their academic lives while remaining aware of its limitations.

However, students’ perceptions varied by socio-demographic and geographic factors. Applied Sciences students value ChatGPT for its technical clarity, while Arts and Humanities students prefer human interaction and express concerns about the tool’s ability to capture nuanced insights. Social Sciences students find ChatGPT limited in providing subjective insights necessary for their disciplines. In low-income regions, students appreciate ChatGPT as essential support where resources are scarce, whereas those in high-income regions focus more on its innovative features and advanced functionalities. Male students report higher satisfaction with ChatGPT, while female students express greater ethical concerns regarding its use, including issues of cheating and privacy. First-year students tend to view ChatGPT as a helpful tool for learning, while advanced students question its reliability and relevance to their more complex academic needs. Online learners benefit more from ChatGPT’s digital alignment with their study practices, finding it easier to integrate into their learning routines, while traditional learners often find it less relevant in face-to-face educational contexts. Additionally, students from urban areas generally utilize ChatGPT more than those in rural settings, where access to technology may be limited. Economic status also plays a role, as students from lower economic backgrounds tend to rely on ChatGPT for support in navigating their academic challenges, highlighting its importance in bridging educational gaps.

The factors related to ChatGPT significantly influence how students use it for tasks such as brainstorming, summarizing, and academic writing. Its ability to simplify complex information encourages frequent use, especially for summarizing, while access to extensive information can aid academic writing but may complicate summarizing due to potential overload. Concerns about privacy and academic integrity often reduce students’ use of ChatGPT, particularly in writing tasks. However, students who recognize the potential for skills development, like improving AI literacy, are more inclined to use it for academic writing, while those who feel it enhances their digital content creation skills tend to use it for brainstorming. Emotional factors also play a role, with curiosity and calmness increasing engagement, particularly in brainstorming activities. Overall, these cognitive, ethical, and emotional elements interact to shape how students engage with ChatGPT in their academic work.

### Implications for practice and policy

The core contribution of the study lies in its comprehensive global analysis of higher education students’ early perceptions regarding the use of ChatGPT. It explores how students initially engage with ChatGPT by identifying the benefits and challenges associated with its use in academic settings, highlighting variations in perceptions based on socio-demographic and geographic factors, and examining how various factors significantly influence student usage. While the results reveal strong student interest and perceived benefits, a deeper critical engagement with these early perceptions, particularly the differences across socio-demographic and geographic characteristics, highlights several significant implications for higher education practice and policy.

The varied perceptions of ChatGPT among students provide valuable insights for higher education practice (i.e., managers and teachers) seeking to implement AI thoughtfully across diverse socio-demographic and geographic contexts. Tailoring ChatGPT to specific disciplines can enhance its educational impact, support technical problem-solving in applied sciences, and foster idea generation in arts and humanities. Customizing AI approaches by income region helps bridge resource gaps by equipping students from low income regions with training for academic support and offering advanced functionalities to high-income students. Gender-based initiatives, such as peer-led tech roles for male students and responsible AI workshops for female students, foster inclusivity and confidence in AI use. First-year students benefit from foundational skills training, while advanced students gain guidance on critically assessing AI content. Integrating ChatGPT as a core tool in online and blended settings and as supplementary support in traditional learning optimizes its adaptability across environments. Addressing geographic and economic distinctions through targeted training ensures ChatGPT becomes a valuable educational resource for rural and economically disadvantaged students while enabling wealthier students to explore more advanced features.

For higher education policy (i.e., policymakers), equitable access to AI in education is essential to prevent the widening of the digital divide. However, paywall restrictions on the latest ChatGPT versions risk leaving students from low income regions and rural areas with outdated tools, limiting the quality of their learning and digital skill development. This gap also affects gender equity, as limited access can hinder female students from building AI literacy and confidence. Addressing these disparities requires funding for subsidized access, institutional licenses, and digital literacy programs in underserved areas, alongside gender-inclusive initiatives like responsible AI workshops. Establishing curriculum guidelines that integrate AI across all learning stages creates a balanced, inclusive approach that enriches traditional education and prepares students for an AI-driven workforce.

### Limitations

Although the study’s large and diverse global sample of students is a notable strength of our research, several limitations must be acknowledged. First, the use of convenience sampling for recruiting participants led to uneven representation across various socio-demographic subgroups, with geographical coverage being a prime example. Despite including participants from over 100 countries and/or territories, less than 1% came from low income countries. Therefore, some findings may be biased to some extent, and caution should be exercised when generalizing the results to countries and/or territories not adequately represented in the sample. Second, the study captured only early impressions and experiences of students with ChatGPT. As generative AI technologies evolve and students become more familiar with their strengths and limitations, these initial impressions may not fully align with their future opinions on ChatGPT. Third, the questionnaire relied on students’ self-reports, which can be subject to information bias. Therefore, it is plausible that some students may have either underestimated or overestimated their early perceptions of ChatGPT in various aspects. Finally, the identified socio-demographic and geographic differences in students’ early perceptions may also reflect factors beyond ChatGPT that were not covered in the questionnaire, such as variations in the digital transformation of higher education, economic development, cultural and religious backgrounds, and political circumstances.

### Future research

Despite the above limitations, our global study is extremely important as it fills a gap in comparative studies analyzing students’ early perceptions of ChatGPT and highlights avenues for future research. First, enhancing sampling methods by employing stratified or random sampling techniques could improve representation across socio-demographic and geographic subgroups, including underrepresented low income countries. Second, conducting longitudinal studies would allow researchers to track changes in students’ perceptions of ChatGPT over time as AI technologies evolve. Third, to counteract potential information bias from self-reports, future studies could incorporate a mix of quantitative and qualitative data sources, such as behavioral data and expert evaluations, alongside self-reports. Finally, investigating a broader range of contextual factors, including the digital transformation of higher education, economic conditions, cultural and religious backgrounds, and political environments, could provide deeper insights into how these factors influence students’ perceptions. These approaches would help build on the current study’s findings and offer a more comprehensive understanding of students’ views on generative AI technologies.

## Supporting information

S1 Checklist Inclusivity in global research. (DOCX)

S1 File Comparative analysis. (XLSX)

S1 Table Spearman correlation between main independent variables of interest. (DOCX)
