# Correction: Higher education students’ perceptions of ChatGPT: A global study of early reactions

**DOI:** 10.1371/journal.pone.0349691

**Published:** 2026-05-20

**Authors:** Dejan Ravšelj, Damijana Keržič, Nina Tomaževič, Lan Umek, Nejc Brezovar, Noorminshah A. Iahad, Ali Abdulla Abdulla, Anait Akopyan, Magdalena Waleska Aldana Segura, Jehan AlHumaid, Mohamed Farouk Allam, Maria Alló, Raphael Papa Kweku Andoh, Octavian Andronic, Yarhands Dissou Arthur, Fatih Aydın, Amira Badran, Roxana Balbontín-Alvarado, Helmi Ben Saad, Andrea Bencsik, Isaac Benning, Adrian Besimi, Denilson da Silva Bezerra, Chiara Buizza, Roberto Burro, Anthony Bwalya, Cristina Cachero, Patricia Castillo-Briceno, Harold Castro, Ching Sing Chai, Constadina Charalambous, Thomas K. F. Chiu, Otilia Clipa, Ruggero Colombari, Luis José H. Corral Escobedo, Elísio Costa, Radu George Crețulescu, Marta Crispino, Nicola Cucari, Fergus Dalton, Meva Demir Kaya, Ivo Dumić-Čule, Diena Dwidienawati, Ryan Ebardo, Daniel Lawer Egbenya, MoezAlIslam Ezzat Faris, Miroslav Fečko, Paulo Ferrinho, Adrian Florea, Chun Yuen Fong, Zoë Francis, Alberto Ghilardi, Belinka González-Fernández, Daniela Hau, Md. Shamim Hossain, Theo Hug, Fany Inasius, Maryam Jaffar Ismail, Hatidža Jahić, Morrison Omokiniovo Jessa, Marika Kapanadze, Sujita Kumar Kar, Elham Talib Kateeb, Feridun Kaya, Hanaa Ouda Khadri, Masao Kikuchi, Vitaliy Mykolayovych Kobets, Katerina Metodieva Kostova, Evita Krasmane, Jesus Lau, Wai Him Crystal Law, Florin Lazăr, Lejla Lazović-Pita, Vivian Wing Yan Lee, Jingtai Li, Diego Vinicio López-Aguilar, Adrian Luca, Ruth Garcia Luciano, Juan D. Machin-Mastromatteo, Marwa Madi, Alexandre Lourenço Manguele, Rubén Francisco Manrique, Thumah Mapulanga, Frederic Marimon, Galia Ilieva Marinova, Marta Mas-Machuca, Oliva Mejía-Rodríguez, Maria Meletiou-Mavrotheris, Silvia Mariela Méndez-Prado, José Manuel Meza-Cano, Evija Mirķe, Alpana Mishra, Ondrej Mital, Cristina Mollica, Daniel Ionel Morariu, Natalia Mospan, Angel Mukuka, Silvana Guadalupe Navarro Jiménez, Irena Nikaj, Maria Mihaylova Nisheva, Efi Nisiforou, Joseph Njiku, Singhanat Nomnian, Lulzime Nuredini-Mehmedi, Ernest Nyamekye, Alka Obadić, Abdelmohsen Hamed Okela, Dorit Olenik-Shemesh, Izabela Ostoj, Kevin Javier Peralta-Rizzo, Almir Peštek, Amila Pilav-Velić, Dilma Rosanda Miranda Pires, Eyal Rabin, Daniela Raccanello, Agustine Ramie, Md. Mamun ur Rashid, Robert A. P. Reuter, Valentina Reyes, Ana Sofia Rodrigues, Paul Rodway, Silvia Ručinská, Shorena Sadzaglishvili, Ashraf Atta M. S. Salem, Gordana Savić, Astrid Schepman, Samia Mokhtar Shahpo, Abdelmajid Snouber, Emma Soler, Bengi Sonyel, Eliza Stefanova, Anna Stone, Artur Strzelecki, Tetsuji Tanaka, Carolina Tapia Cortes, Andrea Teira-Fachado, Henri Tilga, Jelena Titko, Maryna Tolmach, Dedi Turmudi, Laura Varela-Candamio, Ioanna Vekiri, Giada Vicentini, Erisher Woyo, Özlem Yorulmaz, Said A. S. Yunus, Ana-Maria Zamfir, Munyaradzi Zhou, Aleksander Aristovnik

There is an error in affiliation 44 for author MoezAlIslam Ezzat Faris. The correct affiliation 44 is: Faculty of Allied Medical Sciences, Applied Science Private University, Jordan, Jordan.
