# Supplementary material for: Higher education students’ perceptions of ChatGPT: A global study of early reactions
Source: PLoS One. 2025 Feb 5;20(2):e0315011. doi: 10.1371/journal.pone.0315011 (PMC11798494; doi:10.1371/journal.pone.0315011)
Supplement: S1 Table — (DOCX) [file pone.0315011.s003.docx]

Table 1: Spearman correlation between main independent variables of interest

|  | Q19g | Q19f | Q23d | Q21c | Q25b | Q25d | Q26a | Q26b | Q29i | Q28i | Q30e | Q30i | Q32l |
| --- | --- | --- | --- | --- | --- | --- | --- | --- | --- | --- | --- | --- | --- |
| Q19g | 1.000 |  |  |  |  |  |  |  |  |  |  |  |  |
| Q19f | 0.622*** | 1.000 |  |  |  |  |  |  |  |  |  |  |  |
| Q23d | 0.131*** | 0.137*** | 1.000 |  |  |  |  |  |  |  |  |  |  |
| Q21c | 0.061*** | 0.068*** | 0.282*** | 1.000 |  |  |  |  |  |  |  |  |  |
| Q25b | 0.323*** | 0.316*** | 0.178*** | 0.048 | 1.000 |  |  |  |  |  |  |  |  |
| Q25d | 0.282*** | 0.262*** | 0.102*** | 0.036*** | 0.479*** | 1.000 |  |  |  |  |  |  |  |
| Q26a | 0.325*** | 0.315*** | 0.154*** | 0.032*** | 0.433*** | 0.415*** | 1.000 |  |  |  |  |  |  |
| Q26b | 0.350*** | 0.327*** | 0.164*** | 0.045*** | 0.476*** | 0.452*** | 0.646*** | 1.000 |  |  |  |  |  |
| Q29i | 0.260*** | 0.248*** | 0.142*** | 0.083*** | 0.351*** | 0.343*** | 0.365*** | 0.386*** | 1.000 |  |  |  |  |
| Q28i | 0.266*** | 0.245*** | 0.106*** | 0.055*** | 0.338*** | 0.362*** | 0.359*** | 0.379*** | 0.490*** | 1.000 |  |  |  |
| Q30e | 0.204*** | 0.210*** | 0.260*** | 0.166*** | 0.297*** | 0.268*** | 0.287*** | 0.290*** | 0.346*** | 0.287*** | 1.000 |  |  |
| Q30i | 0.253*** | 0.234*** | 0.204*** | 0.115*** | 0.321*** | 0.299*** | 0.328*** | 0.343*** | 0.363*** | 0.338*** | 0.462*** | 1.000 |  |
| Q32l | 0.236*** | 0.218*** | 0.165*** | 0.075*** | 0.371*** | 0.277*** | 0.285*** | 0.329*** | 0.281*** | 0.272*** | 0.268*** | 0.283*** | 1.000 |
| Q32e | 0.192*** | 0.174*** | 0.101*** | 0.014 | 0.238*** | 0.223*** | 0.227*** | 0.257*** | 0.230*** | 0.206*** | 0.187*** | 0.187*** | 0.346*** |

Note: * p < 0.05; ** p < 0.01; *** p < 0.001.
